# Integration of cortical inputs in the lateral hypothalamus is dominated by the medial prefrontal cortex

**DOI:** 10.1113/JP288596

**Published:** 2026-06-16

**Authors:** Lotte J. A. M. Razenberg, Pieter Nicolaas de Greef, Huibert D. Mansvelder, Mahesh Miikael Karnani

**Affiliations:** ^1^ Department of Integrative Neurophysiology, Center for Neurogenomics and Cognitive Research Vrije Universiteit Amsterdam, Amsterdam Neuroscience Amsterdam the Netherlands; ^2^ Institute for Neuroscience and Cardiovascular Research The University of Edinburgh Edinburgh UK

**Keywords:** cortex, hypothalamus, optogenetic circuit mapping, synaptic connectivity

## Abstract

**Abstract:**

The lateral hypothalamus (LH) is a critical brain region orchestrating survival behaviours, including feeding. Its sparse local synaptic connectivity allows long‐range projections to modulate its activity. Some of these projections arise from the cerebral cortex, which is known to influence feeding. However the functional and anatomical organization of cortico‐hypothalamic pathways has remained poorly studied. We used anatomical and optogenetic mapping to show that the medial prefrontal cortex (mPFC) is the strongest cortical input source to the LH, followed by a lateral associative region, including the insular cortex (IC), and the ventral subiculum. Inputs from the mPFC and IC had markedly different synaptic dynamics and were integrated supralinearly. IC input surpassed that of the mPFC in a subpopulation of highly excitable dorsal LH neurons which had a strong h‐current. Input from the mPFC showed selective targeting to LH neurons which project back to the mPFC, suggesting the existence of a direct feedback loop. Overall these results identify a direct prefrontal–hypothalamic pathway which is poised to dominate rapid cortical control of hypothalamic activity.

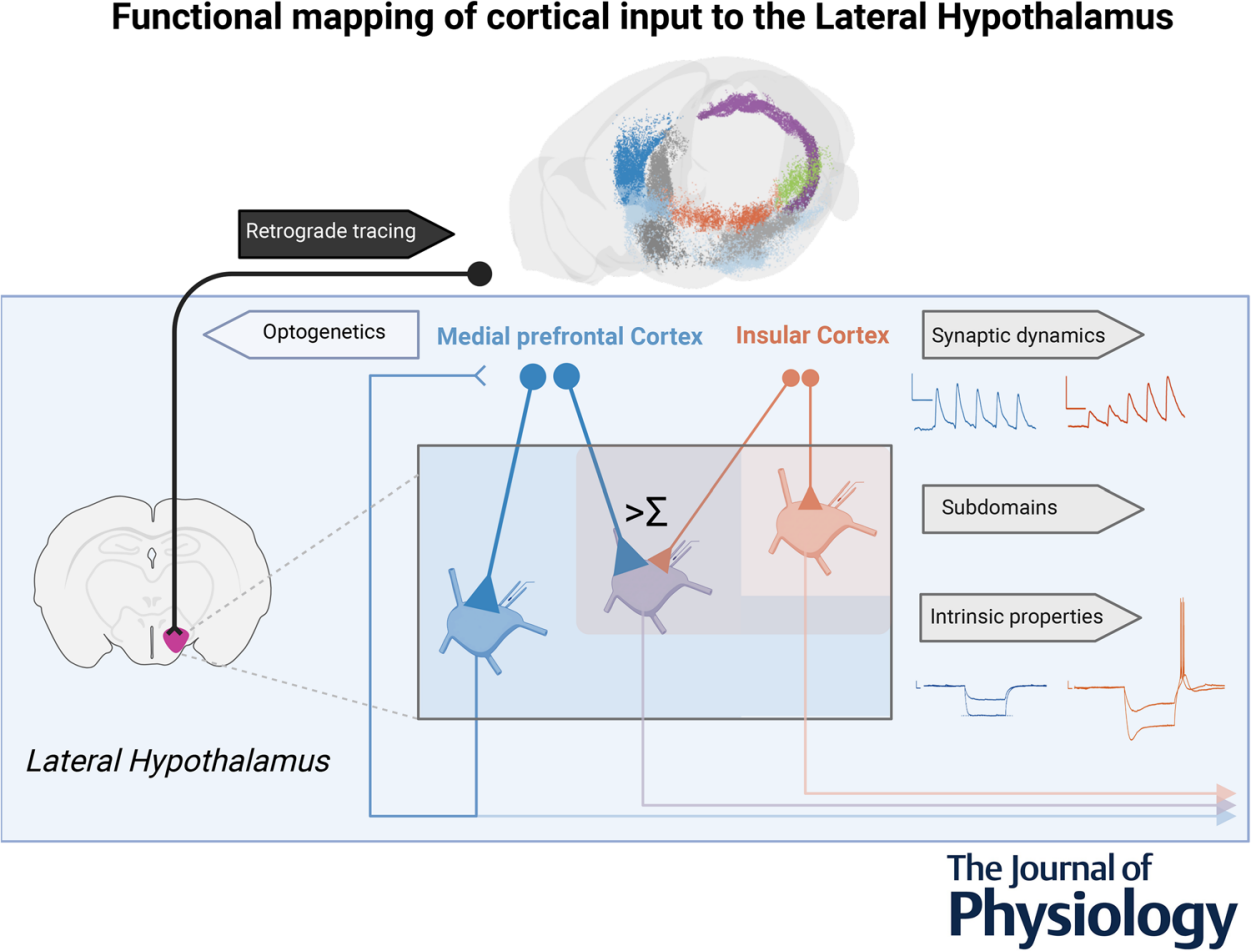

**Key points:**

- The lateral hypothalamus (LH) receives monosynaptic, excitatory cortical synaptic input from associative cortical regions and hippocampus.
- Cortical input is integrated temporally in a pathway‐specific manner within individual LH neurons.
- Inputs from the medial prefrontal cortex (mPFC) and insula are integrated supralinearly.
- The mPFC is a dominant source targeting LH neurons projecting back to the mPFC.

## Graphical abstract

**Abstract figure legend** The lateral hypothalamus (LH) is a critical controller of innate behaviours such as feeding. It is anatomically connected to specific cortical areas. We mapped these areas using a combination of retrograde tracing and anterograde optogenetic circuit mapping. The medial prefrontal and insular cortices are key sources of neocortical output, and they have different synaptic dynamics. Dual‐colour optogenetics revealed that these inputs are integrated supralinearly and are routed to partially distinct LH cells depending on their intrinsic electrophysiological properties. Cortical input from specific associative areas is integrated within individual LH neurons and affects their action potential output.

## Introduction

The lateral hypothalamus (LH) is a critical regulator of feeding, arousal and energy balance (Bonnavion et al., 2016). These functions need to be controlled based on changing internal and external sensory information, which can, in principle, be computed locally or relayed from upstream brain regions. Local computations within the LH are constrained by its ultra‐sparse intrinsic connectivity (Burdakov & Karnani, 2020; Shao et al., 2022), suggesting that its key computation is integration of upstream synaptic inputs within individual neurons. Anatomical studies have established broad connectivity between the LH and many upstream brain regions (Hahn & Swanson, 2012). However the functional properties of these synapses are largely unknown, including their polarity, synaptic strength and temporal as well as spatial integration properties. Therefore a more thorough description of efferents to the LH is needed to understand how it integrates sensory information.

LH neurons are known to have sensory responses (González et al., 2016; Karnani et al., 2020; Rolls, 1984; Siemian et al., 2021), although it is unclear how sensory information is relayed to the LH. The behavioural functions served by the LH are also expected to benefit from control based on predicted future outcomes of actions (Burdakov, 2019; Carpenter, 2004), but it is unknown how predictive information could be computed within the LH. The cerebral cortex is a key site of both sensory and predictive processing (Keller & Mrsic‐Flogel, 2018) and is known to project anatomically to the LH (Haller, 1906; Risold et al., 1997). However the properties of cortico‐hypothalamic pathways have remained poorly characterized.

Given the sparse local synaptic connectivity within the LH (Burdakov & Karnani, 2020; Shao et al., 2022) cortical inputs may strongly influence the firing rates of LH neurons, particularly at rapid behavioural time scales. This may serve multiple behavioural functions, including feeding, which is known to be influenced by frontotemporal cortical regions, including the medial prefrontal cortex (mPFC), insular cortex (IC) and the hippocampus (Baldo et al., 2016; Clarke et al., 2023; Hartmann et al., 2024; Mena et al., 2011; Mohammad et al., 2021; Stevenson & Francis, 2017; Uher & Treasure, 2005; Wu et al., 2020). The effect of sensory and predictive processing on feeding is readily exemplified by detection of food sources and prediction of how much to eat for a given nutritive outcome. The involvement and potential malfunction of the frontotemporal cortices in such behavioural functions are thought to underlie eating disorders (Celeghin et al., 2023; Uher & Treasure, 2005).

The LH contains ∼30 neuronal types (Jiao et al., 2023; Mickelsen et al., 2019), many of which are involved in the control of feeding (Kongstorp et al., 2025). As their firing rates are modulated on a subsecond time scale during feeding (Jennings et al., 2015; Kongstorp et al., 2025; Stamatakis et al., 2016) synaptic excitation of LH neurons by cortical projection neurons may be a key mechanism underlying cortical control of feeding. This is strongly suggested by altered feeding patterns arising from functional manipulation of cortico‐hypothalamic projections (Clarke et al., 2023; Hartmann et al., 2024; Padilla‐Coreano et al., 2022; Supiot et al., 2026). We therefore set out to study the circuit architecture of cortico‐hypothalamic pathways using a combination of retrograde tracing and optogenetic circuit mapping.

We found that cortico‐LH projection neurons were located in frontotemporal associative cortices and ventral subiculum and had a monosynaptic, excitatory effect on LH neurons, whose temporal dynamics depended on the sender region. We used dual‐channel optogenetics to probe the mPFC and IC pathways simultaneously and observed supralinear integration in a subset of LH neurons that receive input from both of these cortical sources. In addition a subpopulation of highly excitable dorsal LH neurons preferentially received IC rather than mPFC input, whereas mPFC input was preferentially targeted at LH neurons, which project back to the mPFC. Our results demonstrate a synaptic basis for control of motivated behaviours, such as feeding, by cortical circuits.

## Materials and methods

### Ethical approval

Ethical approval was obtained from the Dutch Central Authority for Scientific Procedures on Animals (CCD – Centrale Commissie Dierproeven, permit number: AVD11200202114477). All experimental procedures were in accordance with European and Dutch law and approved by the Central Committee on Animal Experiments and the local animal ethical care committee (IvD) of the VU University and VU University Medical Centre (Amsterdam, Netherlands). The authors understand and comply with the animal ethics principles under which the journal operates.

### Animals

Experiments were performed on male and female C57BL/6J mice aged 3–20 weeks. The animals were either obtained from Charles River or were wild‐type breeding surplus from the local breeding facility. All animals were group‐housed with three to four same‐sex littermates and had *ad libitum* access to chow and water. The mice were maintained on a 12:12 light/dark cycle (lights on at 7:00 AM) in a temperature‐ and humidity‐controlled environment.

### Surgeries

To achieve anaesthesia and analgesia mice received an intraperitoneal (i.p.) injection of fentanyl (0.05 mg/kg), medetomidine (0.5 mg/kg) and midazolam (5 mg/kg) in saline. Adequate depth of anaesthesia was confirmed after 10 min by the absence of righting and pedal reflexes. The mice were then placed in a stereotaxic frame on a heated pad, and lidocaine (10 mg/kg) was administered under the scalp for local analgesia. After the skull was exposed small craniotomies were made above the co‐ordinates for the injections (see Table 1, 2 for injection co‐ordinates). Retrograde tracers and/or viruses were diluted in sterile phosphate‐buffered saline (PBS) to eliminate anterograde transsynaptic spread, which was not observed in any of our experiments (see Table 1 for specific concentrations). The injectate was delivered from a glass micropipette using a hydraulic Narishige microinjector at a rate of 10 nl/min for LH injections or 30 nl/min for all other injections. The micropipette was left in place for 25 min after LH injections and 5 min for all other injections. Upon completion of the injections the skin was closed with sutures. Surgical procedures lasted between 45 and 90 min, and anaesthetic depth was assessed by monitoring breathing rate and absence of toe‐pinch reflex. Supplemental anaesthetic doses were not required. To prevent dehydration during prolonged surgeries (>45 min) animals received subcutaneous injections of lukewarm sterile NaCl 0.9% (10 ml/kg) every 45 min. The animals were then removed from the stereotaxic frame and received an i.p. injection of flumazenil (0.5 mg/kg) and atipamezole (2.5 mg/kg) to antagonize the anaesthesia. The mice were placed back into their home cages on a heating pad and allowed to recover. Drinking water was supplemented with carprofen (0.07 mg/ml) 1 day prior to surgery and continued for 3 to 5 days postoperatively, with daily monitoring of body weight, wound healing and general behaviour.

**Table 1:** Injection details

Injectate	Titre/concentration	Volume (nl)	Target area	A‐P (mm from bregma)	M‐L (mm from bregma)	D‐V (mm from brain surface)
Figs 1 and 2						
CTb‐Alexa647/555	1.0 mg/ml	30	LH	−1.3	0.95	−5.4
Figs 3, 4, 5						
AAV1‐hSyn‐ChR2‐YFP	2.3 × 10^12^ vg/ml	100	mPFC (unilateral)	1.8	0.4	−1.9
AAV1‐hSyn‐ChR2‐YFP	2.3 × 10^12^ vg/ml	100	aIC (unilateral)	1	3.2	−2.7
AAV1‐hSyn‐ChR2‐YFP	2.3 × 10^12^ vg/ml	100 total 3 × 33	‐IC‐ (unilateral)	1 0.5 0	3.2 3.7 3.8	−2.7 −2.7 −2.8
AAV1‐hSyn‐ChR2‐YFP	2.3 × 10^12^ vg/ml	100	pIC (unilateral)	0	3.8	−2.8
AAV1‐hSyn‐ChR2‐YFP	2.3 × 10^12^ vg/ml	100	ECT (unilateral)	−3.3	4.3	−2.2
AAV1‐hSyn‐ChR2‐YFP	2.3 × 10^12^ vg/ml	100	dSub (unilateral)	−3.5	2.1	−1.3
AAV1‐hSyn‐ChR2‐YFP	2.3 × 10^12^ vg/ml	100	vSub (unilateral)	−3.8	3.3	−3.2
Figs 6, 7, 8						
AAV1‐Syn‐Chronos‐GFP And/or AAV1‐Syn‐ChrimsonR‐tdT	2.2 × 10^12^ vg/ml 2 × 10^12^ vg/ml	600 total 6 × 100	mPFC (bilateral)	1.8 1.8 2.3	± 0.4 ± 0.4 ± 0.4	−1.9 −1.5 −1.75
AAV1‐Syn‐Chronos‐GFP And/or AAV1‐Syn‐ChrimsonR‐tdT	2.2 × 10^12^ vg/ml 2*10^12^ vg/ml	600 total 6 × 100	IC (bilateral)	1 0.5 0	± 3.2 ± 3.7 ± 3.8	−2.7 −2.7 −2.8
Fig. 9						
AAV1‐hSyn‐ChR2‐YFP CTb‐Alexa647	2.3 × 10^12^ vg/ml 1.0 mg/ml	100 50	mPFC (unilateral)	1.8	0.4	−1.9
AAV1‐hSyn‐ChR2‐YFP CTb‐Alexa647	2.3 × 10^12^ vg/ml 1.0 mg/ml	100 total 3 × 33 50 total 3 × 17	IC (unilateral)	1 0.5 0	3.2 3.7 3.8	−2.7 −2.7 −2.8

*Note*: Anteroposterior (A‐P), mediolateral (M‐L) and dorsoventral (D‐V) injection co‐ordinates in millimetres from bregma per experiment and per target area. All dilutions were made with PBS.

Abbreviations: aIC, anterior insular cortex; d/vSub, dorsal/ventral subiculum; ECT, ectorhinal cortex; ‐IC‐, insular cortex, distributed injection; LH, lateral hypothalamus; mPFC, medial prefrontal cortex; pIC, posterior insular cortex.

### Retrograde tracing

Retrograde tracing was performed using cholera toxin subunit B (CTb) conjugated to Alexa 647 or 555. Labelled cells were quantified semi‐automatically using QuPath (Bankhead et al., 2017). Regions of interest were manually annotated using fiducial landmarks and the Allen Brain Atlas. Cell counting was performed using a combination of manual detection and automatic detection using QuPath's built‐in cell detection function.

**Figure 1:**
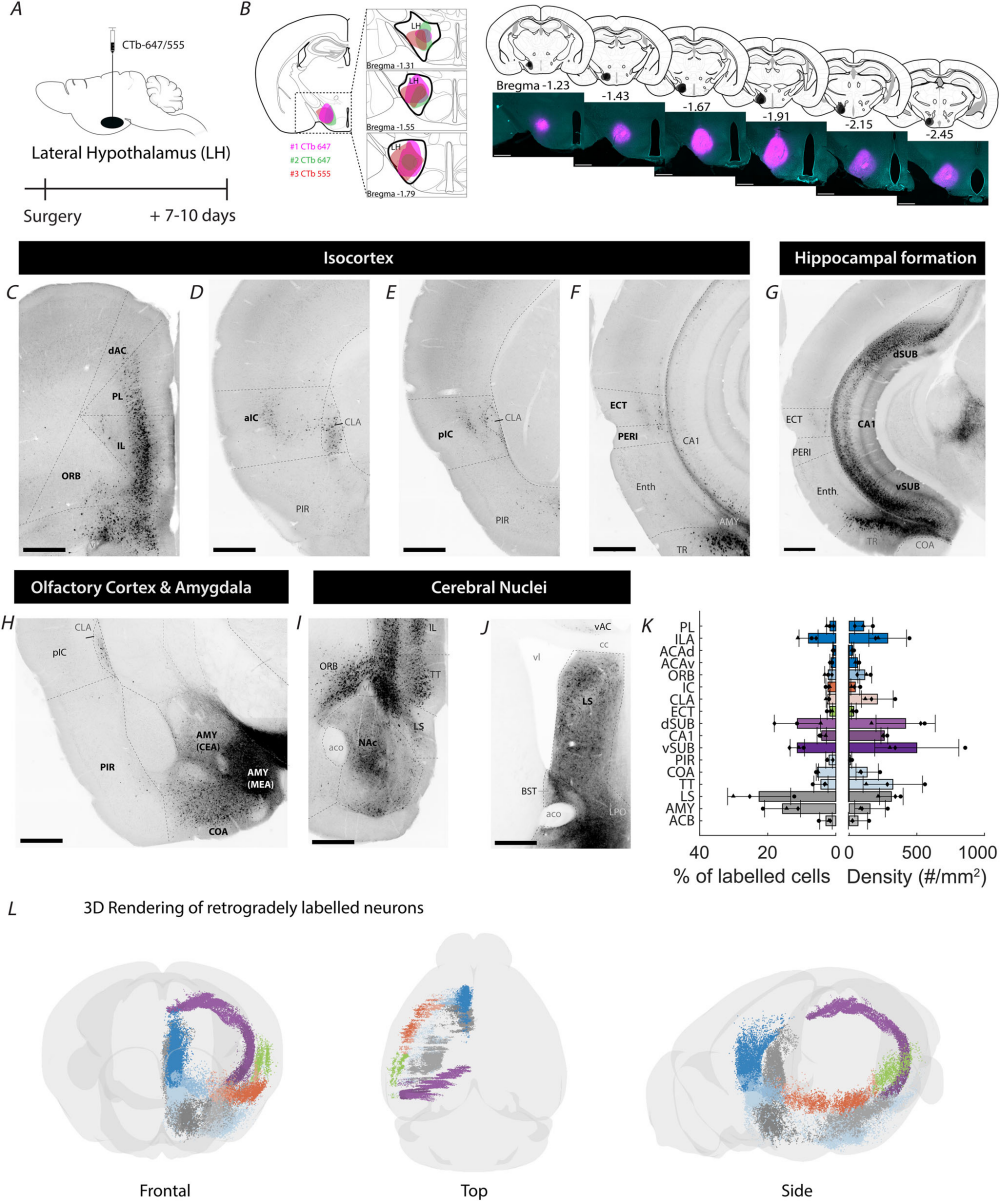
Retrograde tracing from the lateral hypothalamus *A*, schematic of retrograde tracing with cholera toxin subunit B (CTb), conjugated to Alexa‐647 or 555 injected into the lateral hypothalamus (LH). *B*, schematic representations of CTb injections into the LH (*n* = 3 brains), with a representative example. Scalebars represent 500 µm. *C*–*J*, representative images of retrogradely labelled cells in neocortex, hippocampal formation, amygdala, olfactory cortex and cerebral nuclei. Scalebars represent 500 µm. *K*, quantification of retrogradely labelled cells (*n* = 3 brains) per region as a percentage of the total of quantified population (left) and density per area (mm2). Individual data points represent brains, whereas bars and error bars represent the mean and SD (summarized in Table 2). *L*, three‐dimensional (3‐D) views of retrograde labelling (see also Supplementary Video). dAC, dorsal anterior cingulate cortex; aco, anterior commissure; AMY, amygdala; BST, bed nucleus of stria terminalis; CA1, cornu ammonis 1; cc, corpus callosum; CLA, claustrum; COA, cortical amygdala; ECT, ectorhinal cortex; Enth, entorhinal cortex; a/pIC, anterior/posterior insular cortex; IL, infralimbic cortex; LPO, lateral preoptic area; LS, lateral septum; MEA, medial amygdala; NAc, nucleus accumbens; ORB, orbitofrontal cortex; PERI, perirhinal cortex; PIR, piriform cortex; PL, prelimbic cortex; d/vSUB, dorsal/ventral subiculum; TT, taenia tecta; TR, postpiriform transition area; vl, lateral ventricle.

**Figure 2:**
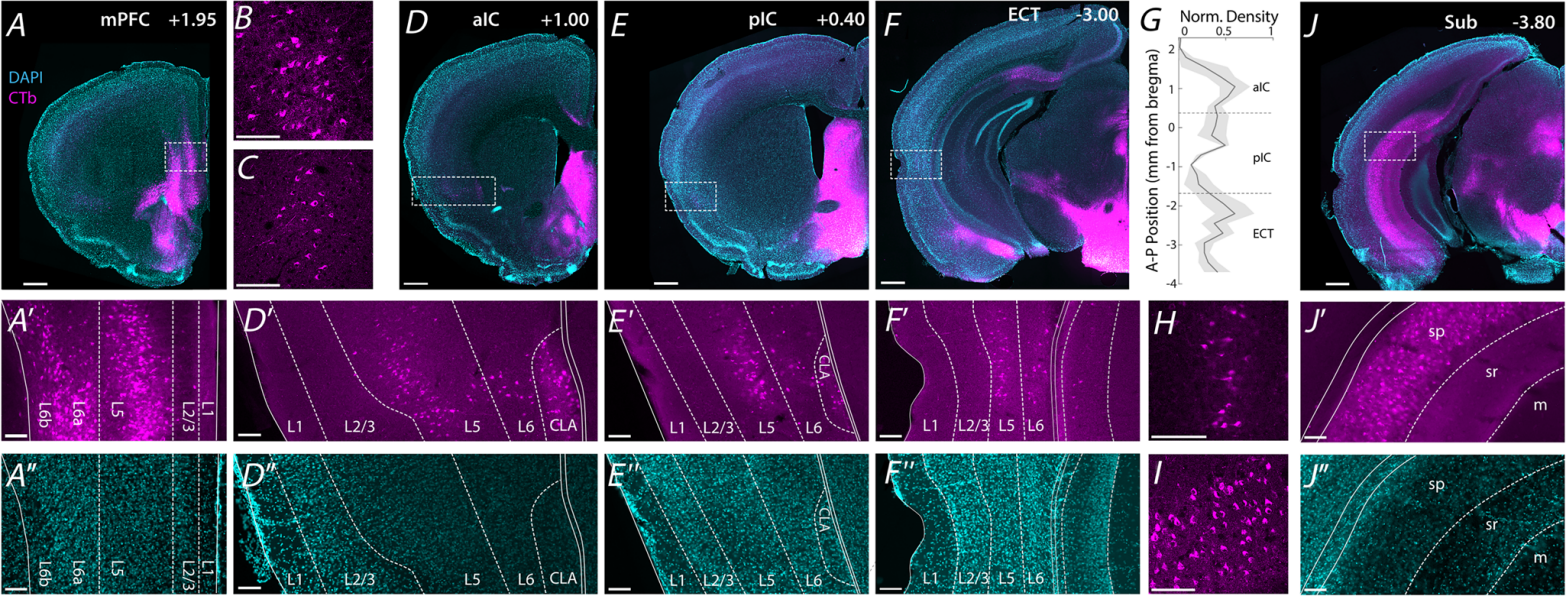
Layer distribution of cortical projection neurons *A*, representative section (1.95 mm anterior from bregma) containing the mPFC. Scalebar represents 500 µm. The bounded box is depicted in A’ (cholera toxin subunit B (CTb)) and A’ (DAPI) to illustrate the distribution of labelled neurons in layers, with scalebars representing 100 µm. *B* and *C*, representative confocal image (40×) of labelled pyramidal neurons in L5 of the mPFC (*B*) and insular cortex (IC) (*C*). *D*–*F*, like (*A*) but for aIC (*D*), pIC (*E*) and ECT (*F*). *G*, normalized density of labelled neurons in lateral associative areas (aIC, pIC, ECT) from anteroposterior bregma +2 mm to −3.8 mm (*n* = 3 brains). Data are represented as means ± SD. *H*, representative image (20×) of labelled pyramidal neurons in L5 of the ECT. *I*, representative confocal image (40×) of labelled pyramidal neurons in the subiculum. *J*, similar to *A* and *D*–*F*, but for the subiculum. A‐P, anteroposterior; mPFC, medial prefrontal cortex; aIC, anterior insular cortex; CLA, claustrum; pIC, posterior insular cortex; ECT, ectorhinal cortex; Sub, subiculum; sp, pyramidal layer; sr, stratum radiatum; m, molecular layer.

For LH retrograde tracing (Figs 1 and 2) three brains were analysed from anteroposterior (A‐P) co‐ordinates 2.23 to −3.79 mm from bregma. Additionally to generate a three‐dimensional (3‐D) model of long‐range projections to the LH one brain was registered to the Allen Brain Atlas using ABBA (Chiaruttini et al., 2025). Detected cells were mapped onto a 3‐D brain model using Brainrender (Claudi et al., 2021).

In combined retrograde and anterograde tracing experiments to characterize reciprocal connections (Fig. 9) between the IC‐LH (*N* = 2 brains) and mPFC‐LH (*N* = 2 brains) retrogradely labelled cells in the LH were quantified in sections spanning −1.07 to 2.06 mm from bregma.

### Anterograde tracing

To quantify the localization and density of axonal fibres in the hypothalamus AAV1‐hSyn‐ChR2‐YFP was injected into either mPFC or IC (see Table 1 for details). Coronal sections containing the LH at A‐P co‐ordinates of −1.07, −1.31, −1.55 and −1.79 mm from bregma were analysed for three brains per injection. We did not observe transsynaptic anterograde‐labelled somata in the LH.

For comparative analysis of fibre distribution imaging parameters were kept consistent across acquisitions, and resulting 8‐bit images were thresholded and binarized using ImageJ to capture labelled fibres while minimizing background signal, using consistent criteria across samples. A custom ImageJ macro was used to align a 20×20 grid onto the LH. The grid origin was defined as the dorsal edge of the third ventricle, and grid dimensions were linearly scaled to span to the lateral edge of the LH and the ventral edge of the section. Thresholded images were measured for integrated density in each bin, and values were exported to MATLAB. Kernel density estimation (KDE) was used to quantify the spatial distribution of bins across mediolateral and dorsoventral axes, evaluated at 100 points per axis. KDE profiles were averaged by A‐P location and injection group, and each sample's peak value was used for statistical comparisons.

### Histology

Tissue was collected 7–10 days after CTb injections and 3–4 weeks after anterograde tracing surgeries to allow for viral expression. Animals were deeply anaesthetized with a terminal dose of pentobarbital (100 mg/kg, i.p., in PBS). Adequate depth of anaesthesia was confirmed after 10–15 min by absence of toe‐ and tail‐pinch reflexes to strong stimuli and absence of visible respiratory movements. Transcardial perfusion with PBS followed by 4% paraformaldehyde (PFA) was then performed, and death occurred as a result of exsanguination during perfusion. Brains were extracted, postfixed in PFA for at least 48 h, sliced at 50–70 µm thickness and mounted using Mowiol mounting medium containing 5 µM 4′,6‐diamidino‐2‐phenylindole (DAPI) for nuclear counterstaining. Slices were imaged using a Vectra Polaris slide scanner (Akoya Biosciences) at 20× resolution.

### Brain slice preparation and solutions

After 3–4 weeks of viral injections mice were anaesthesized with isoflurane (3%–4%, inhalation, delivered in oxygen). Anaesthetic depth was confirmed by absence of toe‐pinch reflex. Mice were then killed by cervical dislocation, and brains were quickly removed following decapitation and placed in ice‐cold *N*‐methyl‐d‐glucamine (NMDG) solution, which included (in mM) the following: 2.5 KCl, 1.2 NaH_2_PO_4_, 30 NaHCO_3_, 20 HEPES, 25 glucose, 5 Na ascorbate, 3 Na pyruvate, 93 NMDG, 10 MgSO_4_ and 0.5 CaCl_2_ (pH 7.3–7.4, 300–310 mOsm). Coronal slices (250 µm thick) containing the LH and injection location were prepared using a vibratome (Leica VT1000 S). The slices were collected and placed in a holding chamber with the NMDG solution maintained at 34°C. After 20 min the slices were transferred to another holding chamber containing ACSF and allowed to recover for at least 1 h at room temperature before recordings. The ACSF solution included (in mM) the following: 126 NaCl, 3 KCl, 2 MgSO_4_, 1.1 NaH_2_PO_4_, 2 CaCl_2_, 10 glucose, 0.1 Na‐pyruvate, 0.4 ascorbic acid, 0.5 glutamine and 26 NaHCO_3_ (pH 7.3–7.4 and 300–315 mOsm). The solutions were continuously bubbled with 95% O_2_ and 5% CO_2_. To verify viral targeting slices containing the injection location were postfixed in 4% PFA for 3–5 days, then mounted with Mowiol mounting medium with 5 µM DAPI for nuclear counterstain. Mice were only included in the analysis if yellow fluorescent protein expression was restricted to the injection location.

### Patch‐clamp recording and analysis

For electrophysiological recordings brain slices were placed in a recording chamber that was continuously perfused with oxygenated ACSF maintained at 32°C. Neurons in the LH were visualized using an Olympus (Tokyo, Japan) BX51WI upright microscope equipped with infrared differential interference contrast optics. Patch pipettes, with a resistance of 3–6 MΩ, were pulled from borosilicate glass capillaries and filled with an internal solution based on potassium gluconate, containing (in mM) the following: 130 K‐gluconate, 5 NaCl, 2 MgSO_4_, 10 HEPES, 0.1 EGTA, 4 MgATP, 0.4 Na‐GTP and 2 pyruvic acid (pH adjusted to 7.3 with KOH). Recordings were performed using Multiclamp 700B amplifiers (Axon Instruments, Molecular Devices). The data were filtered at 3 kHz, digitized at 10 kHz using a National Instruments USB‐6343 digitizer and collected with MIES software (https://github.com/AllenInstitute/MIES) running in IgorPro 8 (WaveMetrics). Recordings were included if access resistance (monitored with a 5 mV hyperpolarizing step between stimulation sweeps) was <25 MΩ and did not increase more than 20% and if the net leak current did not exceed −500 pA.

Input resistance was calculated as the change in membrane potential from baseline to steady‐state divided by the injected current during hyperpolarizing current steps of –50 and –150 pA. Voltage sag was quantified during the –150 pA current step as the difference between the peak hyperpolarization and the steady‐state membrane potential. To account for cell‐to‐cell variability in input resistance and allow more robust comparison across cell populations we also reported the sag amplitude as a percentage of the initial peak deflection. Postinhibitory rebound (PIR) was defined as a depolarization of >5 mV compared to baseline after the −150 pA step.

A current ramp (0–200 pA; duration 15 s) was injected to study potential differences in firing rate. Firing rate was calculated within 8 pA bins (25 bins, 600 ms per bin). Rheobase was defined as the minimal current required to elicit the first action potential. The gain was defined for each neuron as the slope of the linear fit between the bin containing the rheobase and the bin containing the maximum firing frequency.

### Optogenetics

An LED light source (DC4100; Thorlabs) was used to deliver photostimulation light at 470 and/or 590 nm through a 40× objective. For ChR2 stimulation 470 nm irradiance was set at 0.7–1 mW/mm^2^. Cells were current‐clamped around –65 mV. To assess action potentials occurring within these stimulus trains sweeps with action potentials prior to light stimulation were excluded. A 10 Hz train of five light pulses of 3 ms duration was delivered every 15 s to evoke optogenetic responses. At least seven sweeps were averaged to calculate excitatory postsynaptic potential (EPSP) parameters, excluding sweeps with action potentials. The response threshold was set at 4 × SD of a baseline, computed 20 ms before each pulse. EPSP amplitude was computed relative to the baseline prior to each pulse. Responses exceeding the response threshold were considered optically evoked responses and included for further analysis. Latency was defined as time from the light pulse onset to passing the response threshold (set at 4 × SD from baseline). To compare synaptic dynamics of repeated stimulation we analysed the amplitude ratio of the last pulse divided by the first pulse (EPSP5/EPSP1) (Collins et al., 2018).

In experiments using ChrimsonR and/or Chronos opsin expressing neurons at the injection site were current clamped and stimulated with 10 Hz trains of five pulses of 3 ms to get a firing probability across a range of increasing irradiances of 470 and 590 nm light. In recordings from brains with both opsins present potential postsynaptic effects were eliminated with 10 µM DNQX.

In animals only expressing ChrimsonR LH cells were voltage clamped at −70 mV to assess postsynaptic responses across a range of irradiances of 590 and 470 nm light. In subsequent experiments both ChrimsonR and Chronos were expressed in the mPFC and IC of each animal, with injection sites reversed across experiments to control for opsin‐specific effects. After opsin‐specific effects (Fig. 7*E*–*G*) were ruled out recordings were pooled. Irradiances were set at 0.5–0.7 mW/mm^2^ for 590 nm and 0.4–0.6 mW/mm^2^ for 470 nm light. Photostimulation protocols were as follows: (1) 10 ms 590 nm, (2) 10 ms 470 nm, (3) 250 ms 590 nm followed by 10 ms 470 nm (desensitization protocol to suppress ChrimsonR activation by 470 nm light). The sequential photostimulation protocols described above were used to categorize responses in Fig. 7. To compare amplitudes the 590 nm 10 ms light pulse was always compared to the desensitized 470 nm 10 ms light pulse. To study the integration of inputs these individual responses were summed and compared to the amplitude of simultaneous stimulation (10 ms 470 and 590 nm). Integration was quantified as the ratio of dual stimulation to summed responses (dual/sum) for both amplitude and charge transfer. A ratio of 1 indicates linear integration (dual = sum), whereas values >1 indicate supralinear integration (dual > sum). To categorize cells as mPFC‐ or IC‐preferring (Fig. 8) only the desensitization sequence data were used; that is, 250 ms 590 nm pulses were used to evaluate ChrimsonR responses, as this ensured reliable detection across all cells without altering response properties relative to 10 ms stimulation.

In a subset of experiments (Fig. 9) we attempted to either simultaneously or sequentially record CTb+ and CTb− neurons in the same slice. In mPFC‐injected animals 27 cells were recorded from 12 slices in six animals, and in IC‐injected animals 16 cells were recorded from 9 slices in three animals. The average distance between adjacent CTb+ and CTb− cells was 232 ± 139 µm. Across the 43 recorded cells 28 were from slices where both CTb+ and CTb− cells were recorded (16 recorded simultaneously, 12 sequentially), 14 were from the same animals and 1 was a CTb+ cell from a single mPFC‐injected animal. We accounted for variability across cells, slices and animals using a generalized linear mixed model with group as a fixed effect and recording and slice (nested within animal/date) as random effects.

### Pharmacology

Where indicated the voltage‐gated sodium channel blocker tetrodoxin (TTX; 1 µM) was added to the ACSF to inhibit action potentials, along with the voltage‐gated potassium channel blocker 4‐aminopyridine (4‐AP; 250 µM), and DNQX (10 µM) was added to block excitatory connections. All pharmacological agents were bath‐applied for at least 5 min before recordings.

### Locations of recorded neurons

To capture locations of recorded neurons an image of pipette placement within the LH was captured after each recording with a 4× objective. Locations relative to the boundaries of the LH were then calculated using Fiji software and exported to MATLAB by an investigator blinded to the cell identity. In Fig. 8*K* and *L* the shortest distance from the fornix to the dorsal or lateral edge of the LH was used to normalize dorsoventral and mediolateral values, respectively. Normalized values were plotted as fornix = 0, with negative being medial/ventral and positive lateral/dorsal.

### Data analysis and statistics

Data analysis and visualization were performed using custom scripts in ImageJ, MATLAB (2019b), Python and QuPath. Electrophysiological recordings from fewer than five cells are reported qualitatively and were not included in statistical analyses. All statistical analyses were performed in MATLAB, and detailed descriptions of the specific statistical tests are provided in Statistical Summary.

Normality was assessed using the Shapiro–Wilk test before statistical testing. For data that did not meet normality assumptions the following non‐parametric tests were applied: Wilcoxon signed‐rank test, Wilcoxon rank sum test and Kruskal–Wallis test. Multiple comparisons were corrected using the false discovery rate (FDR) method, and adjusted *P*‐values are reported. Proportional data, such as response rates, were analysed using χ^2^, as indicated in text, and followed up using Fisher's exact tests. Generalized linear mixed‐effects models (GLMEs) were applied to non‐normally distributed multifactorial datasets and analyses involving repeated measures (e.g. accounting for opsin and cortical area effects on inputs in dual‐channel experiments or comparing responses to consecutive pulses). Model selection was based on visual inspection of data distributions to identify the appropriate distribution family (e.g. Gamma), along with model performance evaluation using the Akaike information criterion and residual analysis.

All data are presented as individual datapoints along with means ± SD, unless otherwise specified. Statistical significance is indicated in figures using the following notation: *P* < 0.05 (*), *P* < 0.01 (**) or *P* < 0.001 (***).

## Results

### Retrograde tracing identifies a continuum of associative cortical L5/L6 neurons as potential sources of LH input

To explore which cortical populations project to the LH we injected the retrograde tracer CTb conjugated to Alexa 647 or 555 into the LH (Fig. 1*A*,*B*; *N* = 3). We observed substantial retrograde labelling in the neocortex (Fig. 1*C*–*F*), hippocampus (Fig. 1*G*), as well as the amygdala (AMY, Fig. 1*H*), nucleus accumbens (Nac, Fig. 1*I*) and lateral septum (LS, Fig. 1*J*). Within the neocortex extensive labelling was observed in the mPFC, predominantly in the infralimbic (IL) and prelimbic (PL) areas, with sparser labelling in the anterior cingulate (AC, Fig. 1*C*). The density of labelling in IL and PL was comparable to that observed in the LS, suggesting that neocortical inputs to the LH may be as substantial as inputs from subcortical input hubs like the LS (Fig. 1*K*).

**Table 2:** Retrograde labelling (related to Fig. 1)

		% of labelled cells	Density (cells/mm^2^)
Area	*n*	Mean SD	Mean SD
PL	3	1.70 ± 1.00	107.03 ± 66.58
IL	3	8.01 ± 2.80	284.73 ± 139.44
dAC	3	0.77 ± 0.14	26.27 ± 8.17
vAC	3	1.37 ± 0.37	60.81 ± 14.41
ORB	3	2.21 ± 1.05	115.06 ± 50.87
IC	3	2.30 ± 0.60	45.59 ± 33.00
CLA	3	2.34 ± 0.47	208.05 ± 116.70
ECT	3	1.75 ± 0.80	33.74 ± 18.04
dSub	3	11.28 ± 0.68	417.05 ± 219.02
CA1	3	4.13 ± 1.38	259.62 ± 24.91
vSub	3	11.30 ± 2.12	499.50 ± 310.89
PIR	3	1.97 ± 0.97	10.20 ± 9.21
COA	3	5.42 ± 0.33	132.76 ± 82.55
TT	3	4.37 ± 2.08	322.47 ± 219.90
LS	3	22.53 ± 9.27	311.66 ± 87.08
AMY	3	15.65 ± 5.26	154.13 ± 113.22
NAc	3	2.89 ± 1.83	64.93 ± 70.94

Abbreviations: AMY, amygdala; CA1, cornu ammonis 1; CLA, claustrum; COA, cortical amygdala; d/vAC, dorsal/ventral anterior cingulate cortex; d/vSUB, dorsal/ventral subiculum; ECT, ectorhinal cortex; IC, insular cortex; IL, infralimbic cortex; LS, lateral septum; NAc, nucleus accumbens; ORB, orbitofrontal cortex; PERI, perirhinal cortex; PIR, piriform cortex; PL, prelimbic cortex; TT, taenia ecta.

The overall population of labelled neurons in the neocortex was continuous across anatomical boundaries and adjacent coronal sections. To capture this we generated a 3‐D rendering from one hemisphere (Fig. 1*L*, see Supplementary Video). From the mPFC the labelled population extended ventrally into the taenia tecta (TT) and dorsal peduncular area (DP; Fig. 1*C*,*I*) and caudally into the LS (Fig. 1*J*). The labelled population continued laterally from the IL, DP and TT into orbital areas (ORB, Fig. 1*I*), extending further into the claustrum (CLA) and the IC (Fig. 1*D*,*E*,*H*), and caudally to the ectorhinal cortex (ECT, Fig. 1*F*–*G*). Notably cortical labelling was restricted to associative areas, and no labelling was detected in primary sensory or motor cortices. The continuous labelling suggests that cortical control of the LH may be co‐ordinated across neocortico‐LH projection neurons connected through local microcircuits.

Further similarities across neocortico‐LH projection cells were their restriction to layers 5 and 6 and pyramidal morphology (Fig. 2). Hippocampal neurons were in the pyramidal layer of the dorsal subiculum (dSub), extending ventrally to include the ventral subiculum (vSub) and the CA1 situated between them (Fig. 1*G* and Fig. 2*F*,*I*,*J*). These anatomical findings set up an expectation of similar excitatory responses in LH neurons receiving input from the different associative cortical regions.

### Functional mapping of cortical synaptic input to LH

**Figure 3:**
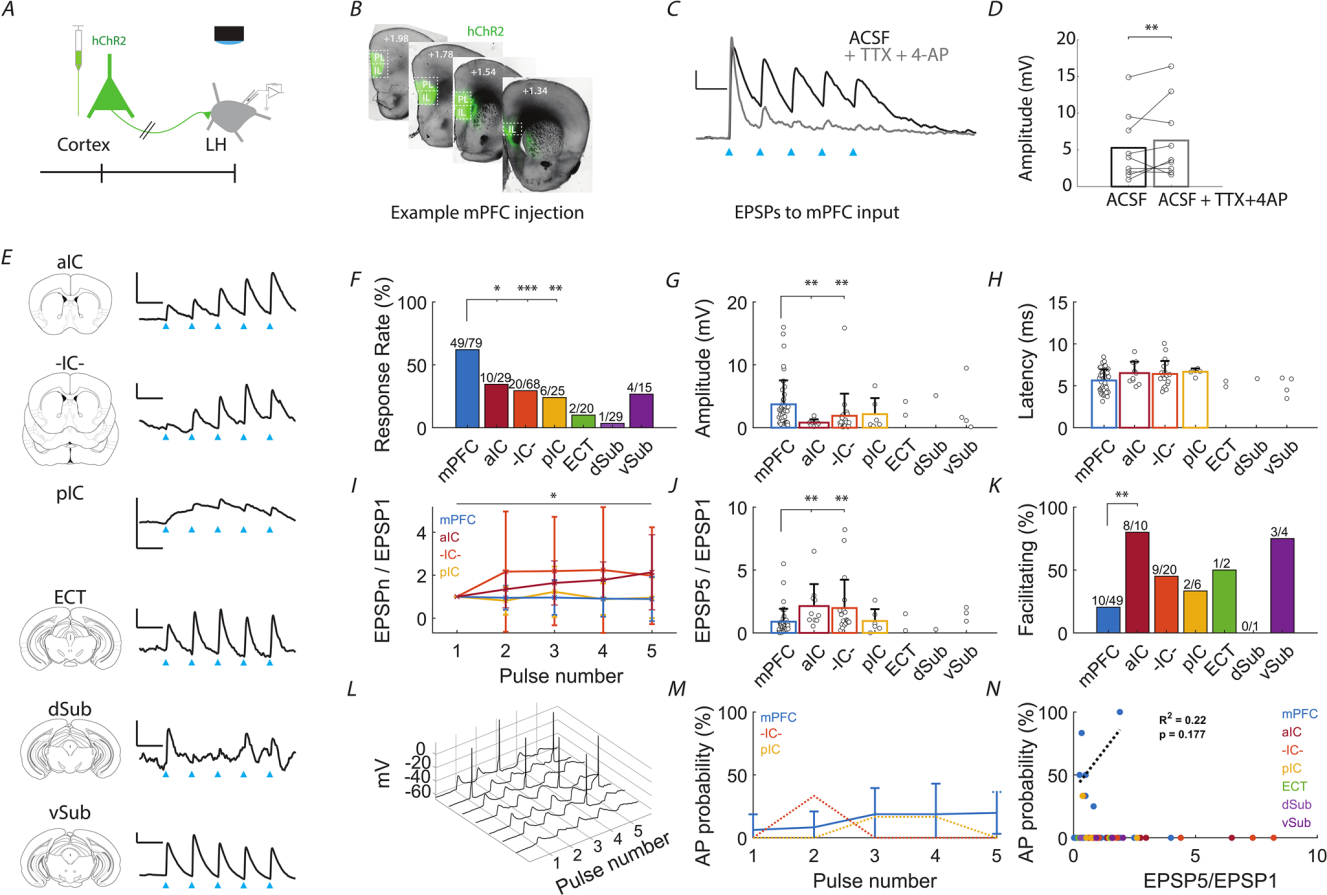
Functional mapping of cortical inputs *A*, schematic of optogenetic circuit mapping in lateral hypothalamus (LH) slices. *B*, representative example of viral expression in mPFC. *C*, example current‐clamp recording in an LH neuron in response to mPFC axon stimulation in baseline (black) and TTX+4‐AP (grey). Scalebars represent 3 mV and 100 ms. *D*, response amplitudes of mPFC‐responsive neurons in baseline (ACSF) and TTX+4‐AP. *E*, schematic and example recording to responses from cortical areas besides mPFC. Scalebars represent 3 mV and 100 ms. *F*, proportions of LH neurons with postsynaptic responses to photostimulation of cortical axons as a percentage of all recordings (mPFC: 49/79 cells, 48 slices, 19 animals; aIC: 10/29, 9 slices, 4 animals; ‐IC‐: 20/68, 27 slices, 10 animals; pIC: 6/25, 17 slices, 6 animals; ECT: 2/20, 6 slices, 2 animals; dSub: 1/29, 13 slices, 6 animals; vSub: 4/15, 7 slices, 3 animals). Statistics were performed on input regions with at least five responsive neurons. mPFC, aIC, ‐IC and pIC showed different response rates (*P* = 0.00009, χ^2^), which were higher for the mPFC than the insular regions (aIC: *P* = 0.032, IC: *P* = 0.0007, pIC: *P* = 0.003, Fisher's exact test). *G*, response amplitude to the first pulse of the stimulus trains differed across cortical input areas (*P* = 0.001, Kruskal–Wallis test). Particularly mPFC inputs were larger than aIC or ‐IC‐ inputs (*P* = 0.004 and *P* = 0.007, Wilcoxon rank sum test). *H*, onset latency of light‐evoked EPSPs (pulse 1). The response latencies were similar across cortical input areas (*P* = 0.066, Kruskal–Wallis test). *I*, summary of paired‐pulse ratio for each light pulse (*n*) in the stimulus train (10 Hz, 3 ms pulses) per input area. A generalized linear mixed‐effects model (GLME) revealed a significant interaction of pulse number and input area (*P* = 0.014, GLME). *J*, the paired pulse ratio of the last and first light pulse (EPSP5/EPSP1) in the stimulus train differed between input areas (*P* = 0.001, χ^2^). aIC and ‐IC‐ EPSPs were more facilitating than mPFC EPSPs (*P* = 0.006; *P* = 0.010, Fisher's exact test). *K*, percentage of responsive cells with facilitating responses per input area. Responses were considered facilitating if EPSP5/EPSP1 was larger than 1. The proportion of facilitating responses differed per input area (*P* = 0.002, χ^2^), and pairwise comparisons revealed that facilitating responses to aIC inputs were significantly more frequent compared to mPFC inputs (10/49 *vs*. 8/10; *P* = 0.004, Fisher's exact test) *L*, example AP firing in response to photostimulation of mPFC axons. *M*, summary of the probability of AP firing for each light pulse in the stimulus train per input area. A GLME revealed no significant effects of cortex, pulse number or their interaction on AP probability (see Statistical Summary). *N*, scatter plot of EPSP5/EPSP1 and the probability of firing at least one AP during the stimulus train. Individual dots represent cells, coloured by injection area. Dashed line shows the linear regression of cells with AP probability > 0 (R2 = 0.22, *P* = 0.177). AP, action potential; mPFC, medial prefrontal cortex; aIC, anterior insular cortex; ‐IC‐, insular cortex, distributed injection; pIC, posterior insular cortex; ECT, ectorhinal cortex; dSub, dorsal subiculum; vSub, ventral subiculum.

Retrograde tracers are taken up at axons terminating in the injected area but also by fibres of passage. We therefore assessed functional synaptic connectivity by expressing channelrhodopsin‐2 in one cortical region at a time, allowing for photostimulation of their axon terminals in acute LH slices (Fig. 3*A*,*B*). We divided the IC into anterior (aIC) and posterior (pIC) regions, as they have distinct projection patterns (Gehrlach et al., 2020) and performed an additional multi‐injection across the A‐P extent to encompass the full IC projection population (‐IC‐). We performed these recordings across the following sample sizes of cells/slices/animals: mPFC: 79/48/19; aIC: 29/9/4; ‐IC‐: 68/27/10; pIC: 25/17/6; ECT: 20/6/2; dSub: 29/13/6; vSub: 15/7/3.

Robust light‐evoked EPSPs were recorded in whole‐cell current clamp (Fig. 3*C*–*E*), and these responses were resistant to a combination of tetrodotoxin and 4‐aminopyridine (TTX+4‐AP, mPFC: *n* = 9/9, Fig. 3*C*,*D*; IC: *n* = 6/6; vSub: *n* = 4/4), indicating direct glutamatergic synapses (Petreanu et al., 2009), as expected for a presynaptic population composed of pyramidal neurons. Pooled responses in TTX+4‐AP were 62% larger than in baseline (3.9 ± 4.7 mV *vs*. 6.4 ± 6.8 mV, *n* = 19, *P* = 0.001, Wilcoxon signed‐rank test). Onset latencies of the subset of neurons confirmed to be monosynaptic using TTX+4‐AP were not different from those of all other recorded responses (ACSF latencies: 5.8 ± 1.3 ms *vs*. 6.0 ± 1.4 ms; *P* = 0.829, Wilcoxon rank sum test).

A higher proportion of LH neurons (49/79) responded to inputs from the mPFC than any of the other areas (10/29 aIC, 20/68 ‐IC‐, 6/25 pIC, 2/20 ECT, 1/29 dSub, 4/15 vSub, Fig. 3*F*). Statistical comparisons, for subsequent analyses excluding groups with fewer than five responding neurons, confirmed that mPFC inputs recruited a significantly higher proportion of neurons than aIC (*P* = 0.032), ‐IC‐ (*P* = 0.0007) or pIC (*P* = 0.003, Fisher's exact tests). Inputs from dSub were notably sparse (1/29, Fig. 3*F*), despite extensive retrograde labelling (Figs. 1 and 2), suggesting very strong convergence or retrograde tracer take‐up by fibres of passage.

Response amplitudes to the first pulse were significantly larger for mPFC than IC EPSPs (3.7 ± 3.8 mV, *n* = 49 *vs*. 1.9 ± 3.5 mV, *n* = 20, *P* = 0.007, Wilcoxon rank sum test, Fig. 3*G*), whereas maximum EPSP amplitude during 10 Hz stimulus trains was not different (4.3 ± 3.9 mV *vs*. 2.7 ± 3.6 mV, *P* = 0.091, Wilcoxon rank sum test). Onset latencies were not significantly different across cortical input regions (*P* = 0.066, Kruskall–Wallis test, Fig. 3*H*). IC responses were significantly more facilitating than mPFC responses (EPSP5/EPSP1 ratio: 2.0 ± 2.3 *vs*. 0.9 ± 1.0, *P* = 0.010, Wilcoxon rank sum test, Fig. 3*I*–*K*). In terms of IC subdivisions aIC EPSPs had particularly low first pulse responses (0.8 ± 0.5 mV, *n* = 10, *P* = 0.004 compared to mPFC, Wilcoxon rank sum test, Fig. 3*G*) and high facilitation (2.1 ± 1.8, *P* = 0.006, compared to mPFC, Wilcoxon rank sum test, Fig. 3*E*,*I*–*K*). These data point to an A‐P gradient of increasingly facilitating LH output synapses within the lateral neocortex (ECT‐aIC), distinct from the mostly non‐facilitating mPFC output (Fig. 3*J*,*K*).

Action potential firing, though rare overall, was only observed in response to mPFC and IC stimulation (Fig. 3*L*,*M*); however given the low numbers of recorded cells we cannot rule out the possibility of other cortical regions inducing spiking in LH. GLME revealed no significant effects of cortical region (*P* = 0.552) on action potential probability, and the probability of firing at least one action potential during the stimulus train was not significantly different across inputs (12% mPFC, 1.9% IC, 6.7% pIC, 0.0% aIC, *P* = 0.251, Kruskal–Wallis test). Action potential probability was not correlated with the EPSP5/EPSP1 ratio (*P* = 0.177, linear regression, Fig. 3*N*), suggesting that synaptic facilitation may not strongly affect action potential output.

### Topographical overlap of cortical axons and responsive neurons in LH

**Figure 4:**
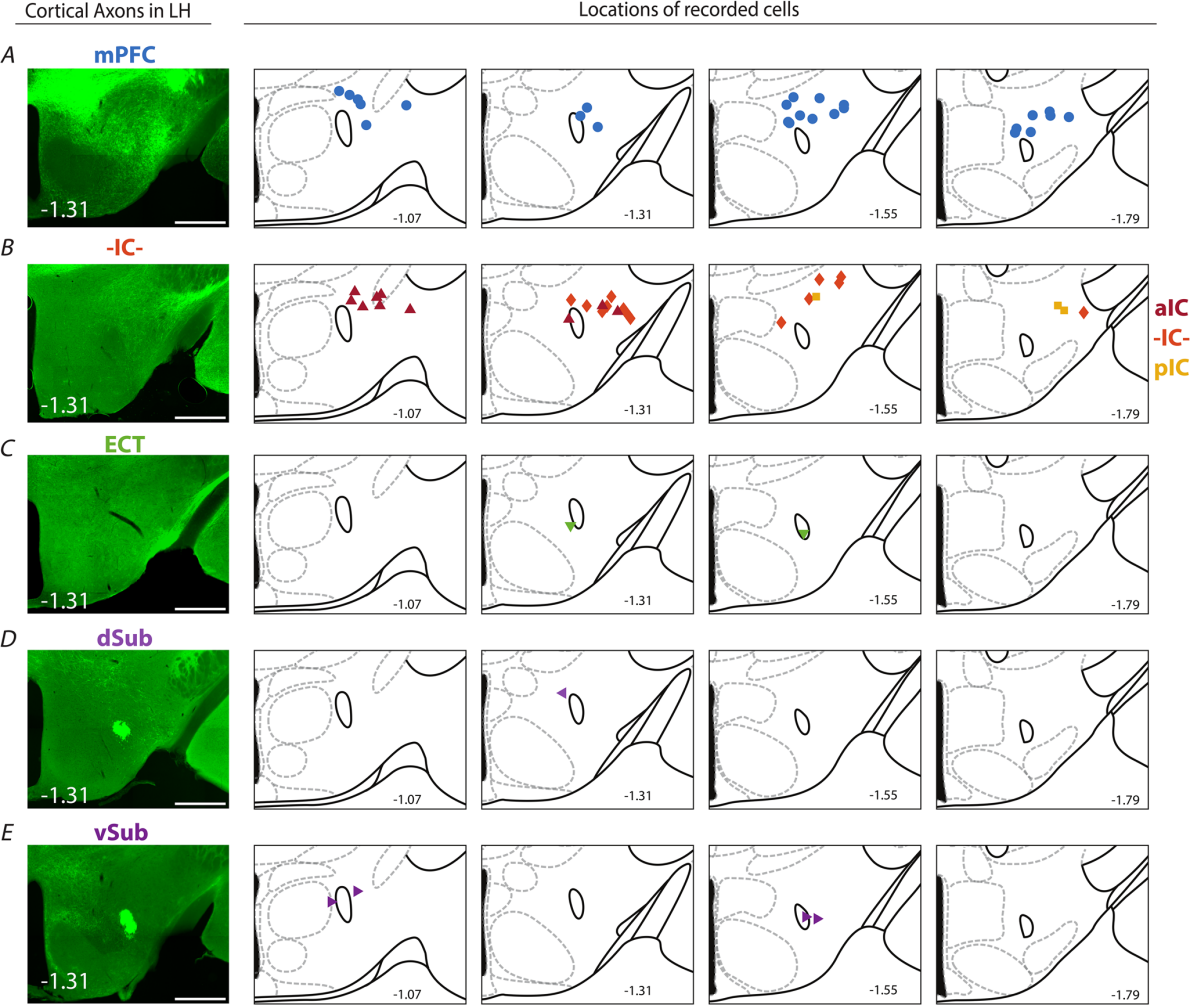
Anatomical locations of cortical input–responsive LH neurons *A*–*E*, left: YFP‐labelled cortical axons in representative LH sections (bregma – 1.31 mm). Scalebars represent 500 µm. Right: normalized locations of responding neurons (from Fig. 3). LH, lateral hypothalamus; mPFC, medial prefrontal cortex; aIC, anterior insular cortex; ‐IC‐, insular cortex, distributed injection; pIC, posterior insular cortex; ECT, ectorhinal cortex; dSub, dorsal subiculum; vSub, ventral subiculum.

We next asked if cortical inputs target overlapping subregions of the LH. To address this we compared the spatial distribution of cortical axons and locations of recorded neurons. Anterograde tracing suggested that axons from different cortical regions are differentially distributed within the LH and aligned with the locations of postsynaptic neurons (Fig. 4). Axons from the mPFC and IC were abundant, whereas ECT, dSub and vSub axons appeared sparser (Fig. 4), consistent with the observed response rates (Fig. 3*F*). Notably dSub axons were primarily localized in the fornix, consistent with the possibility that the dense retrograde labelling arose from take‐up by fibres of passage (Figs 1 and 2).

**Figure 5:**
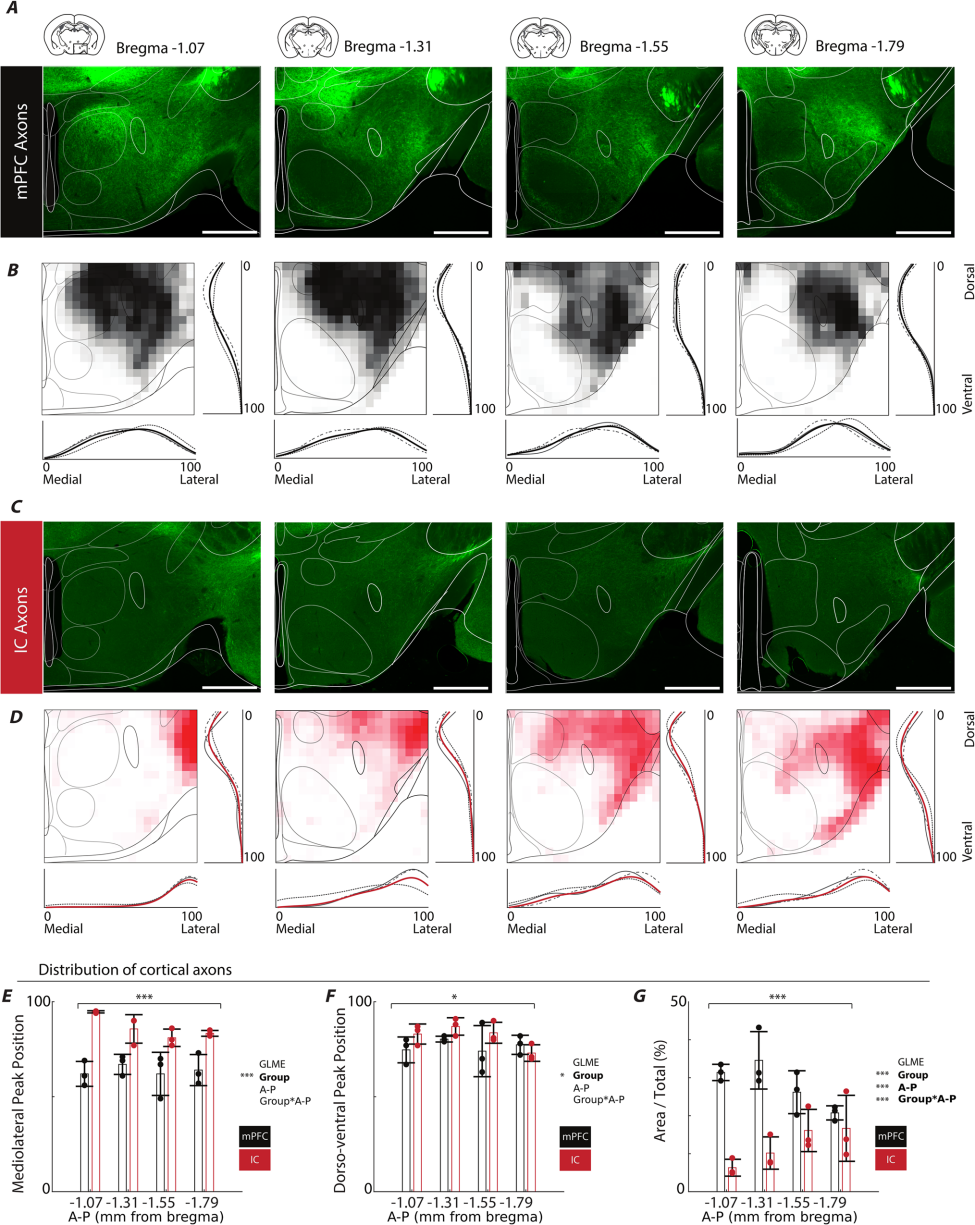
Topographical organization of frontal cortical axons in LH *A*, representative examples of anterograde‐labelled mPFC axons in LH in four different anteroposterior (A‐P) positions, ranging from −1.07 to −1.79 mm from bregma. *B*, heatmaps of averaged mPFC axonal densities across three samples, measured on a 20 × 20 grid (see Methods). Kernel density estimation (KDE) profiles along the mediolateral and dorsoventral axes are shown for individual samples (dashed lines) and the average (solid thick line). KDE was evaluated at 100 points per axis. The *x*‐axis (0–100) represents normalized dorsal‐to‐ventral or medial‐to‐lateral positions, and the *y*‐axis shows KDE. *C* and *D*, same as *A* and *B* but for IC axons (*n* = 3). *E*, mediolateral peak position of the KDE for mPFC and IC axons across four different A‐P position. GLME revealed a significant effect of group (mPFC *vs*. IC,  *P* = 0.0001), but not A‐P position (*P* = 0.278) or their interaction (*P* = 0.094). *F*, dorsoventral peak position of the KDE for mPFC and IC axons across four A‐P positions. GLME revealed a significant effect of group (*P* = 0.031) but not A‐P position (*P* = 0.298) or their interaction (*P* = 0.127). *G*, proportion of total area covered by mPFC and IC axons across A‐P positions. GLME revealed significant effects of group (*P* <0.0001), A‐P position (*P* = 0.001) and their interaction (*P* = 0.0001). mPFC, medial prefrontal cortex; IC, insular cortex; GLME, generalized linear mixed‐effects model.

To quantitatively assess topographical organization we performed additional anterograde tracer injections in mPFC and IC (*N* = 3 animals per group) and analysed fibre distributions across the mediolateral (M‐L) and dorsoventral (D‐V) axes using KDE. IC and mPFC axons were concentrated in largely overlapping regions of LH (Fig. 5*A*–*D*), suggesting that they can reach overlapping pools of LH neurons.

Comparisons using GLME, accounting for repeated measurements across A‐P positions within animals, revealed that despite this overlap IC axons were positioned more laterally (M‐L peak of KDE: 86 ± 7 *vs*. 64 ± 7, *P* = 0.0001) and dorsally (D‐V peak of KDE: 18 ± 7 *vs*. 23 ± 7, *P* = 0.031) than mPFC axons (Fig. 5*E*,*F*). Conversely mPFC projections occupied a larger overall fraction of the LH than IC projections, although this difference varied across A‐P positions (28 ± 7% *vs*. 12 ± 7%, *P* = 0.0001, GLME, Fig. 5*G*). These findings suggest that although different cortical regions contribute uniquely to LH innervation, mPFC and IC inputs may target the partially overlapping pools of LH neurons.

### Supralinear integration of mPFC and IC inputs in LH neurons

**Figure 6:**
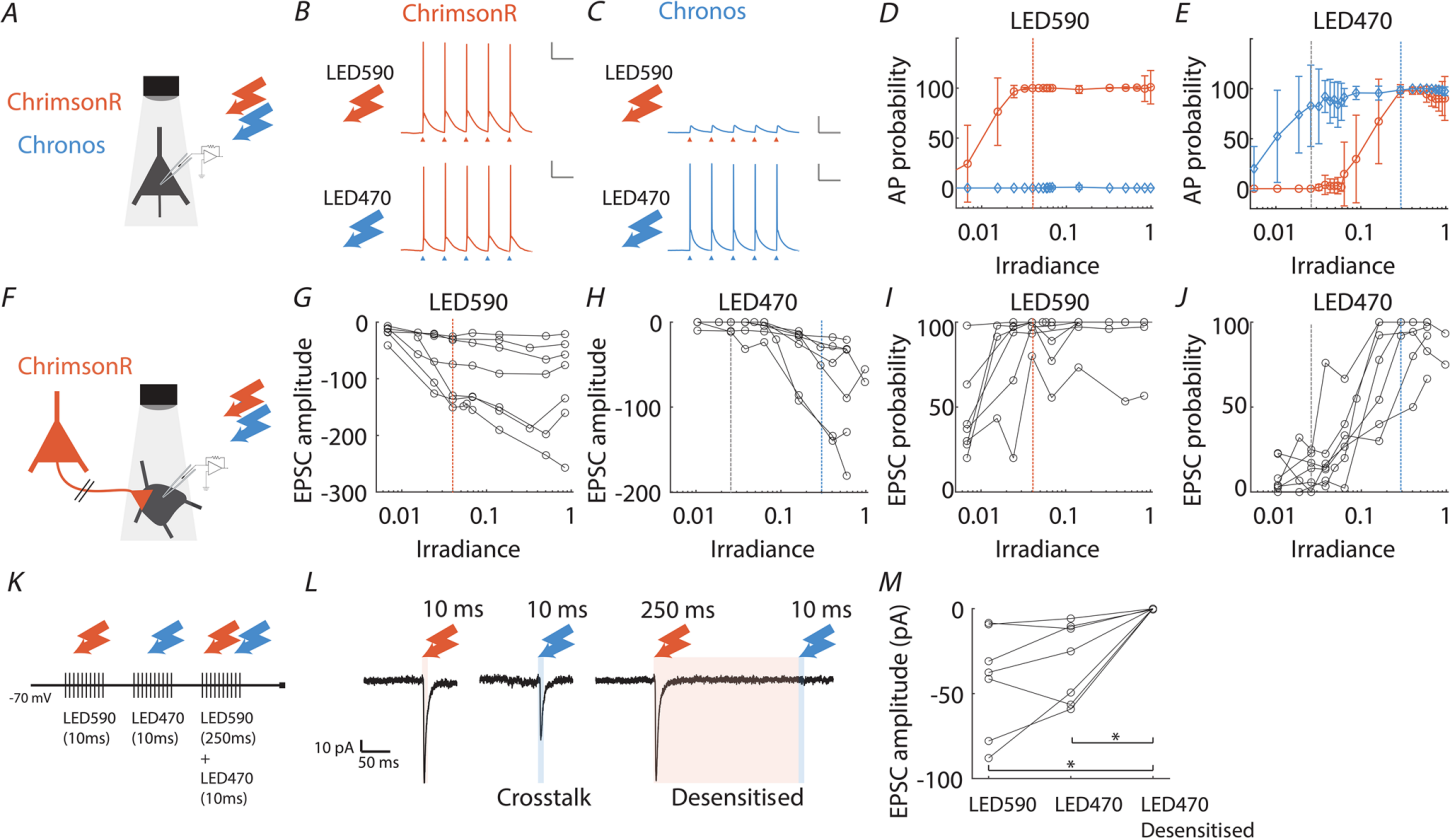
Verification and optimization of dual‐channel optogenetics strategy *A*, schematic of photostimulation and recording strategy. ChrimsonR‐ or Chronos‐expressing neurons at the injection site were recorded to assess light sensitivity to 470 nm (blue arrow) and 590 nm (orange arrow). ChrimsonR: *n* = 8 cells, 6 slices, 5 animals; Chronos: *n* =   6 cells, 4 slices, 3 animals. *B*, example traces of a Chrimson‐expressing neuron at −68 mV resting membrane potential. Light stimulation (five pulses of 3 ms at 10 Hz), with 590 nm light induced reliable firing with a minimal irradiance of 0.016 mW/mm2 (top) and 470 nm light induced firing with a minimal irradiance of 0.088 mW/mm2 (bottom). *C*, same as *B* but for a Chronos‐expressing neuron at −65 mV resting membrane potential. The 590 nm light did not induce firing even at maximal power of 1 mW/mm2 (top), and the 470 nm light induced firing with a minimal irradiance of 0.025 mW/mm2 (bottom). Scalebars in *B* and *C* represent 20 mV and 100 ms. *D*, AP probability in ChrimsonR‐ (orange) or Chronos (blue)‐expressing neurons for different irradiances of 590 nm light. The dashed orange line indicates minimum irradiance for 100% ChrimsonR AP probability. *E*, same as *D* but for photostimulation with 470 nm light. The dashed blue line indicates minimum irradiance for 100% Chronos A‐P probability. The minimum irradiance needed for 100% of Chronos neurons (0.29 mW/mm2) is indicated by a blue dashed line and for the example in C (bottom) by a grey dashed line. *F*, schematic of LH recordings in *G–J*. *G*, EPSC amplitude in response to 590 nm light of increasing irradiance. Connected dots represent measurements from individual cells (*n* = 7 cells, 3 slices, 2 animals). *H*, same as *G* but for 470 nm light. *I*, EPSC probability in response to photostimulation with 590 nm light of increasing irradiance. *J*, same as *I* but for 470 nm light. *K*, schematic representation of photostimulation protocols in subsequent recordings. A stimulation block of 10 ms pulses of 590 nm light was followed by a block of 10 ms pulses of 470 nm light, and finally a block of 250 ms 590 nm followed directly by 10 ms 470 nm to desensitize ChrimsonR. Each block contained 10 sweeps with a 10 s intersweep interval. *L*, example traces of a postsynaptic LH neuron, averaged per stimulus block, showing that crosstalk stimulation of ChrimsonR terminals by 470 nm light is suppressed by the desensitization protocol. *M*, EPSC amplitudes in response to 10 ms stimulation with 590 nm light and to 470 nm light (crosstalk), and to 470 nm light preceded by a desensitizing 590 nm light pulse (LED590 *vs*. LED470, *P* = 0.128; LED590 *vs*. LED470 desensitized, *P* = 0.027; LED470 *vs*. LED470 desensitized, *P* = 0.027, Wilcoxon signed‐rank test). EPSC, excitatory postsynaptic current.

We next set up a dual‐channel optogenetics strategy to directly test if individual LH neurons integrate mPFC and IC input. Each cortical area expressed either the blue‐light‐activated opsin, Chronos or the red‐light‐activated ChrimsonR (Klapoetke et al., 2014). To validate this approach we first virally expressed ChrimsonR or Chronos in separate animals (Fig. 6*A*) and recorded action potential firing in opsin‐expressing neurons at the injection site in response to 470 and 590 nm stimulation across a range of light intensities (ChrimsonR: *n* = 8 cells, 6 slices, 5 animals; Chronos: *n* = 6 cells, 4 slices, 3 animals; Fig. 6*B*–*E*). As expected 590 nm light stimulation selectively evoked action potentials in ChrimsonR‐expressing neurons but not in those expressing Chronos (Fig. 6*B*–*D*). However the 470 nm irradiance required to achieve 95%–100% action potential probability in Chronos‐expressing neurons also induced action potentials in ChrimsonR‐expressing neurons (Fig. 6*B*,*C*,*E*). We also tested if EPSCs were evoked by 470 and 590 nm stimulation across a range of light intensities in LH neurons of animals only expressing ChrimsonR (*n* = 7 cells, 3 slices, 2 animals; Fig. 6*F*–*J*) and found that 470 nm stimulation readily evoked EPSCs (Fig. 6*H*,*J*). However the selective activation range without triggering ChrimsonR action potentials was suboptimal, with a firing probability of only 83 ± 16% in Chronos‐expressing neurons, which still induced small EPSCs in the LH of ChrimsonR animals with up to 25% probability (Fig. 6*J*). Therefore we used sequential photostimulation to desensitize ChrimsonR with a 250 ms 590 nm pulse prior to a high‐intensity 470 nm pulse to selectively drive Chronos (Fig. 6*K*–*M*), which effectively eliminated ChrimsonR EPSCs in response to 470 nm light (−31 ± 23 pA before *vs*. −0.1 ± 0.2 pA after desensitization pulse, *n* = 7, *P* = 0.027, Wilcoxon signed‐rank test) (Bauer et al., 2021; Shelton et al., 2025).

**Figure 7:**
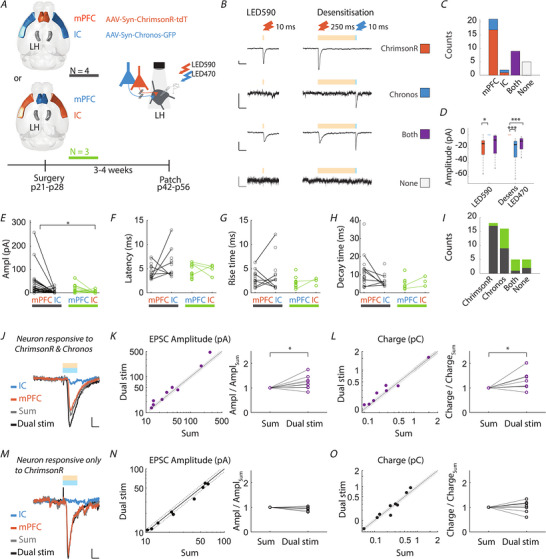
mPFC and IC inputs converge supralinearly in LH *A*, schematic of dual‐channel optogenetics (see Table 1 for injection details). Mice were injected with both AAV1‐Syn‐ChrimsonR‐tdT and AAV1‐Syn‐Chronos‐GFP bilaterally into the mPFC and the IC (top, *N* = 4 animals), or vice versa (bottom, *N* = 3), to account for opsin effects. *B*, example postsynaptic responses to 10 ms 590 nm light, 10 ms 470 nm light after desensitization of ChrimsonR with 250 ms 590 nm light (see Fig. 6). Response categories from top to bottom: response exclusively to ChrimsonR, response exclusively to Chronos, responses to both opsins, no response to either opsin. Scalebars represent 10 pA and 50 ms. *C*, cell count of neurons pooled from both injection groups (*N* = 44 cells, from 26 slices, 7 animals), with responses to exclusively mPFC input (*n* = 21/44), exclusively IC input (*n* = 2/44), mPFC and IC input (both, *n* = 16/44) and of neurons with no EPSC to either input (none, *n* = 5/44). *D*, summary of EPSC response amplitudes per response category coloured as in *B* and *C*. (590 nm: ChrimsonR neurons *n* = 18 *vs*. Chronos neurons *n* = 5, *P* = 0.034, Wilcoxon rank sum test. Desensitized 470 nm: ChrimsonR neurons *n* = 18 *vs*. Chronos neurons *n* = 5,  *P* = 0.001; ChrimsonR *vs*. both *n* = 16, *P* < 0.0001, Wilcoxon rank sum test).  *E*–*H*, characteristics of mPFC and IC responses, grouped by opsin combination (mPFC‐ChrimsonR and IC‐Chronos in black, *n* = 29 cells, in 16 slices, 4 animals; or mPFC‐Chronos and IC‐ChrimsonR in green, *n* = 15 cells, 10 slices, 3 animals) to control for opsin effects on EPSC amplitude (*E*) (absolute values), latency (*F*), rise time (*G*) and decay time (*H*). Linear mixed‐effects analysis revealed a significant effect of cortical area, but not opsin, on amplitude (*P* = 0.0495), but not on latency (*P* = 0.462), rise time (*P* = 0.708) or decay time (*P* = 0.181). Recorded responses are shown as individual data points with lines connecting responses from the same cell. *I*, absolute cell counts for opsin response categories, showing contributions per injection group. (mPFC‐ChrimsonR and IC‐Chronos in black; or mPFC‐Chronos and IC‐ChrimsonR in green). *J*, overlaid traces of a representative neuron integrating cortical responses from IC (blue, 470 nm after desensitization pulse) and mPFC (orange), the sum of the mPFC and IC responses (grey) and simultaneous dual‐colour stimulation (black). *K*, Left: summed amplitudes of mPFC and IC EPSCs compared against those from simultaneous dual‐colour stimulation in cells that had convergent inputs. Diagonal in black and ± 10% as dotted lines. Right: dual stimulation amplitudes divided by the summed amplitudes to assess linearity (*n* = 10, *P* = 0.038, Wilcoxon signed rank test). Scalebars represent 10 pA and 5 ms. *L*, same as *K* but for the integral of the EPSC, which represents total synaptic charge transfer (pC). The ratio for dual stimulation is larger than 1 (*P* = 0.037, Wilcoxon signed‐rank test). *M*–*O*, same as *J*–*L* but for a subset of neurons that responded exclusively to ChrimsonR. *M*, overlaid traces of representative neuron with response to mPFC (ChrimsonR, orange) but not IC (Chronos, blue). Scalebars represent 10 pA and 5 ms. *N*, comparison of summed *versus* dual stimulation response amplitudes (*n* = 9, *P* = 0.066, Wilcoxon signed‐rank test) across recordings as in *M*. *O*, comparison of total synaptic charge transfer of summed *versus* dual stimulation responses (*P* = 0.575, Wilcoxon signed‐rank test). mPFC, medial prefrontal cortex; IC, insular cortex; Ampl, amplitude.

We then expressed ChrimsonR and Chronos in the mPFC and IC using bilateral, equal‐volume injections to cover projection populations, reversing injection sites across experiments to control for opsin‐related effects (Fig. 7*A*; *n* = 44 cells, from 26 slices, in 7 animals). Applying the desensitization strategy described above LH neurons were classified as responsive to ChrimsonR (response to 590 nm light), Chronos (no response to 590 nm light but a response to 470 nm light after desensitization), both or neither (Fig. 7*B*–*D*). We further confirmed that ChrimsonR 470 nm responses were eliminated by the desensitization strategy (−19 ± 14 pA before *vs*. 0.0 ± 0.0 pA after desensitization pulse, *n* = 18, *P* < 0.0001, Wilcoxon signed‐rank test, see Statistical Summary). Most neurons received input from the mPFC, either exclusively or in combination with IC inputs (*n* = 37/44, 84%). Of these 36% (16/44) integrated inputs from both the mPFC and IC, whereas 48% (21/44) received input exclusively from the mPFC, and only 4% (2/44) received input exclusively from the IC. The occurrence of cells with both mPFC and IC inputs did not significantly differ from a chance overlap (*P* = 0.88, χ^2^), consistent with independent targeting by mPFC and IC inputs.

We compared EPSC parameters using GLME, controlling for the effects of opsin type (Chronos *vs*. ChrimsonR), injection site (mPFC *vs*. IC) and their interaction (Fig. 7*E*–*I*, see Statistical Summary for details). EPSCs evoked by mPFC stimulation exhibited significantly larger amplitudes than IC EPSCs (−25 ± 45 pA *vs*. −3.6 ± 7.3 pA, *P* = 0.0495, Fig. 7*E*), whereas latencies (4.0 ± 1.8 ms *vs*. 5.2 ± 3.2 ms, *P* = 0.462, Fig. 7*F*), rise times (2.4 ± 1.5 ms *vs*. 3.3 ± 3.3 ms, *P* = 0.708, Fig. 7*G*) and decay times were similar (8.2 ± 7.1 ms *vs*. 6.0 ± 4.1 ms, *P* = 0.181, Fig. 7*H*).

We next asked if LH neurons receiving convergent inputs from both the mPFC and IC integrated these inputs linearly. To do this we compared the summed EPSCs to simultaneous dual‐colour stimulation (Fig. 7*J*). Simultaneous dual‐colour stimulation produced larger response amplitudes than the calculated sum (121 ± 27%, *n* = 10, *P* = 0.038, Wilcoxon signed‐rank test, Fig. 7*K*) and greater synaptic charge transfer (126 ± 35%, *P* = 0.038, Fig. 7*L*). These data indicate supralinear summation of the mPFC and IC input in LH neurons.

To rule out the possibility that supralinear summation results from increased excitability of ChrimsonR‐expressing axons during dual‐colour stimulation we performed the same analysis in LH neurons that received input exclusively from ChrimsonR‐positive cortical axons (ChrimsonR‐only neurons; Fig. 7*M*–*O*). In this subset simultaneous dual‐colour stimulation did not produce larger responses than the calculated sum (amplitude: 95 ± 8%, *n* = 9, *P* = 0.066, Wilcoxon signed‐rank test; charge: 104 ± 22%, *n* = 9, *P* = 0.575, Wilcoxon signed‐rank test, Fig. 7*M*–*O*) or red light alone (amplitude: 101 ± 9%, *n* = 9, *P* =   0.594, Wilcoxon signed‐rank test). This is consistent with our earlier control experiments demonstrating that EPSC amplitudes are saturated at the higher light intensities (Fig. 6*G*–*J*). This control demonstrates that the supralinear summation effect is specific to neurons receiving convergent input from both cortical sources and does not arise from artefactual opsin coactivation.

### Neurons preferentially responsive to IC input are highly excitable and clustered in dorsal LH

**Figure 8:**
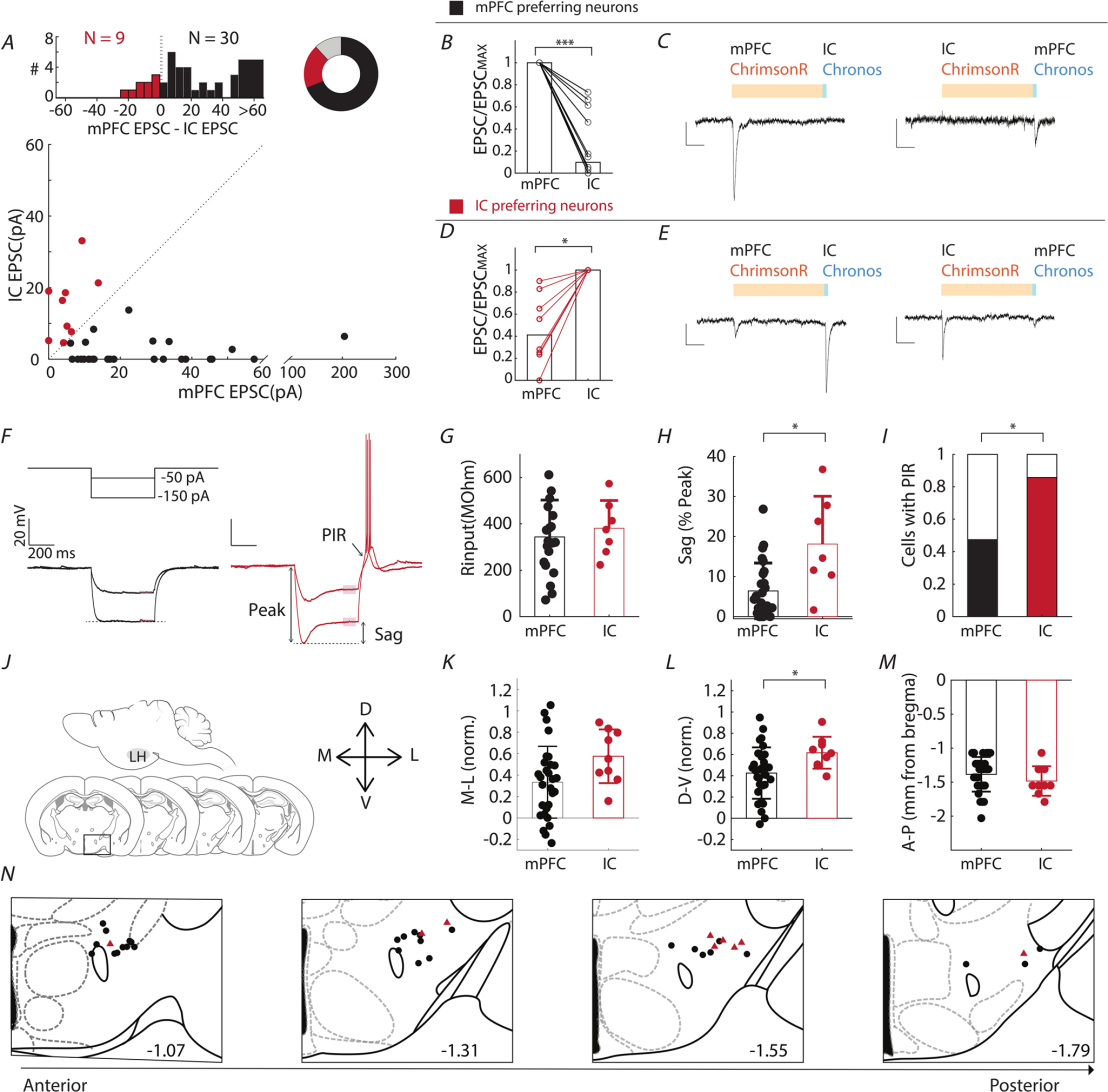
Increased excitability and dorsal location of IC‐preferring LH neurons *A*, (bottom) amplitudes of EPSCs in LH neurons to evoked mPFC and IC input in dual‐channel optogenetics (Fig. 7). The dashed line is the diagonal, where mPFC and IC inputs would be equal.  *A*, (top left) histogram of the difference between mPFC and IC EPSC amplitudes. Neurons with larger mPFC EPSC than IC EPSC (black, *n* = 30) were considered ‘mPFC‐preferring’, whereas the rest were considered ‘IC‐preferring’ (red, *n* = 9). (*A*, top right): pie chart summarizing the total of 44 neurons recorded (68% mPFC‐preferring, *n* = 30/44, black; 20% IC‐preferring, *n* = 9/44, red; 11% non‐responsive, *n* = 5/44, grey). *B*, mPFC‐preferring neuron EPSCs normalized to peak response (*P* < 0.0001, Wilcoxon signed‐rank test). *C*, example traces of mPFC‐preferring neurons using two different combinations of opsin. ChrimsonR was stimulated by a 250 ms pulse of 590 nm light, followed by a 10 ms pulse of 470 nm light to stimulate Chronos. *D* and *E*, same as *B* and *C*, but for IC‐preferring neurons *n* = 9 (*P* = 0.008, Wilcoxon signed‐rank test). Scalebars in *C* and *E* represent 10 pA and 10 ms. *F*, example voltage responses to negative current steps (−150 and −50 pA), for a mPFC‐preferring neuron (black) and IC‐preferring neuron (red). Scalebars correspond to 20 mV and 200 ms. *G*, input resistance (Rinput) for both input types (*P* = 0.491, Wilcoxon rank sum test).  *H*, voltage sag was calculated as the percentage of the initial peak deflection to account for variation in Rinput (*P* = 0.038, Wilcoxon rank sum test). *I*, fraction of neurons displaying a postinhibitory rebound (PIR, *P* = 0.044, χ^2^). *J*, schematic overview of slice locations, ranging anteroposteriorly from −1.07 to −1.79 mm from bregma. *K*–*M*, Summaries of cell locations. Dorsoventral and mediolateral co‐ordinates were normalized to edge markers and plotted as fornix = 0, with negative being medial/ventral and positive being lateral/dorsal. *K*, mediolateral locations (M‐L, *P* = 0.075, Wilcoxon rank sum test), (L) dorsoventral locations (D‐V, *P* = 0.017, Wilcoxon rank sum test) and (M) anteroposterior locations (A‐P, *P* = 0.193, Wilcoxon rank sum test). *N*, plots of relative locations of neurons, where black dots represent PFC‐preferring neurons, and red dots represent IC‐preferring neurons. mPFC, medial prefrontal cortex; IC, insular cortex; EPSC, excitatory postsynaptic current; PIR, postinhibitory rebound; Rinput, input resistance; LH, lateral hypothalamus; D, dorsal; L, lateral; V, ventral; M, medial; M‐L, mediolateral; D‐V, dorsoventral; A‐P, anteroposterior.

Although across all our recordings mPFC input was on average stronger than IC input (Figs. 3*G* and 7*E*), a subset of neurons had stronger IC input (Fig. 7*C*,*E*). We therefore asked if these distinctly sensitive neurons represent an electrophysiologically distinguishable subtype (Karnani et al., 2013). We categorized neurons based on their dominant input type (mPFC‐preferring or IC‐preferring) and compared their intrinsic properties (Fig. 8*A*–*I*). Both types of neurons had similar input resistances (mPFC‐preferring: 343 ± 159 MΩ, *n* = 21 *vs*. IC‐preferring: 381 ± 120 MΩ, *n* = 7, *P* = 0.491, Wilcoxon rank sum test, Fig. 8*G*), but IC‐preferring neurons showed a more pronounced voltage sag in response to hyperpolarizing current steps (4.4 ± 4.7 mV *vs*. 11.7 ± 9.1 mV, *P* = 0.033, Wilcoxon rank sum test, Fig. 8*F*,*H*) and a higher incidence of postinhibitory rebound depolarization (10/21 *vs*. 6/7 neurons, *P* = 0.044, χ^2^, Fig. 8*I*). To account for cell‐specific differences in input resistance voltage sag was normalized to the initial peak deflection, which preserved the group difference (7.9 ± 6.9% *vs*. 18.1 ± 12%, *P* = 0.039, Fig. 8*H*). Current ramp measurements revealed no differences in rheobase (30 ± 28 pA, *n* = 16 *vs*. 44 ± 47 pA, *n* = 5, *P* = 0.483, Wilcoxon rank sum test), gain (firing rate/injected current: 0.3 ± 0.2 Hz/pA *vs*. 0.3 ± 0.2 Hz/pA, *P* = 0.650, Wilcoxon rank sum test) or maximal firing rate (35 ± 18 Hz *vs*. 35 ± 19 Hz, *P* = 0.967, Wilcoxon rank sum test). These results suggest that only voltage sag and PIR were increased in IC‐preferring neurons, which likely result from increased h‐current density.

As cortical neurons with voltage sag receive increased excitatory synaptic input (Lee et al., 2014; Popescu et al., 2017; Yamamuro et al., 2018) we analysed spontaneous EPSCs impinging on IC‐preferring and mPFC‐preferring neurons but found no differences in their amplitudes (31 ± 25 pA *vs*. 22 ± 6 pA, *P* = 0.811, Wilcoxon rank sum test) or frequencies (5.7 ± 6.5 Hz *vs*. 5.1 ± 4.4 Hz, *P* = 0.730, Wilcoxon rank sum test), suggesting that cortical input sensitivity and voltage sag are independent of excitatory synaptic activity. Consistent with IC axon organization (Fig. 5) IC‐preferring neurons were located more dorsally within the LH than mPFC‐preferring neurons (normalized D‐V locations 0.44 ± 0.24 *vs*. 0.63 ± 0.15, *P* = 0.017, Wilcoxon rank sum test, Fig. 8*J*–*N*).

mPFC preferentially targets reciprocally connected LH neurons

**Figure 9:**
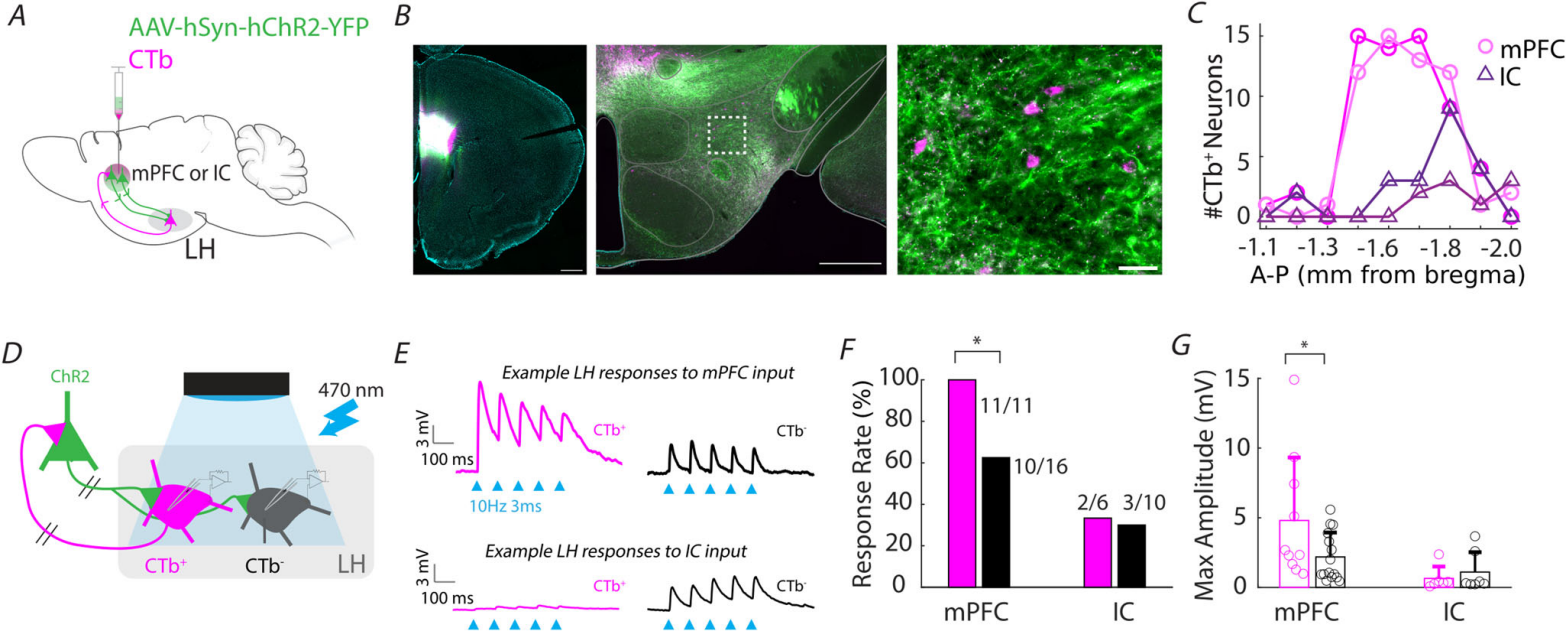
mPFC afferents preferentially target reciprocally connected LHA neurons *A*, schematic of combined injection of AAV‐hSyn‐hChr2‐YFP and retrograde tracer CTb‐647 into the mPFC or the IC to study reciprocal connectivity with the LH. See Table 1 for injection details. *B*, histological sections. Left: example of an injection of CTb‐647 and ChR2‐YFP in the mPFC. Scalebar represents 500 µm. Middle: ChR2‐YFP expressing afferents (green) and retrogradely labelled cell bodies (magenta) in the LH. Scalebar represents 500 µm. Right: high‐resolution micrograph of dashed box in the middle. Scalebar represents 50 µm. *C*, CTb+ neurons counted across LH sections for mPFC injections (magenta, *N* = 2 brains) and IC injections (purple, *N* = 2 brains). Individual brains are plotted as connected markers. *D*, schematic optogenetic recordings after injections into mPFC (*N* = 6 animals) or IC (*N* = 3 animals). CTb+ and CTb− neurons in the LH were recorded during photostimulation with 470 nm light (5 pulses of 3 ms at 10 Hz). *E*, example traces of CTb+ (reciprocal) and CTb− (non‐reciprocal) neurons in the LH responding to mPFC (top) or IC (bottom) input with EPSPs. *F*, response rates for mPFC and IC injections. The proportion of optogenetically responsive CTb+ (magenta) and CTb− neurons (black). mPFC: 100%, 11/11 neurons *versus* 67%, 10/16 neurons, *P* = 0.033, χ^2^, *n* = 27 cells, 12 slices, 6 animals. IC: 33%, 2/6 neurons *versus* 30%, 3/10 neurons, 16 cells, 9 slices, 3 animals; no statistical comparisons were made between IC categories due to low response numbers. *G*, EPSP amplitudes of CTb+ and CTb− neurons responding to mPFC or IC input (*P* = 0.011, GLME). mPFC, medial prefrontal cortex; IC, insular cortex; LH, hypothalamus; CTb, cholera toxin subunit B; GLME, generalized linear mixed‐effects model; A‐P, anteroposterior.

The LH projects to various downstream and upstream targets, and previous tracing work has suggested the LH is connected reciprocally with most of these targets (Hahn & Swanson, 2012; Hahn et al., 2022). We therefore asked whether mPFC and IC inputs are biased towards reciprocally connected neurons. To investigate this we co‐injected ChR2 and CTb into either the mPFC or the IC (Fig. 9*A*). This reliably retrogradely labelled neurons in the LH, with a tendency for more neurons retrogradely labelled from the mPFC than IC (117 *vs*. 30, counted from 9 slices from 2 mice/sample, Fig. 9*B*–*C*).

We then performed whole‐cell current clamp recordings in retrogradely labelled neurons to assess their responsiveness to cortical input; 100% (11/11) of retrogradely labelled LH→mPFC neurons received mPFC input compared to 63% of non‐mPFC‐projecting neurons (10/16), a significant difference (*N* = 6 mice, *P* = 0.033, χ^2^, Fig. 9*D*–*F*). IC axon stimulation elicited similar response rates in LH→IC and non‐IC‐projecting neurons (33% (2/6) *vs*. 30% (3/10), *N* = 3 mice, *P* = 0.889, χ^2^, Fig. 9*G*). Given the lower response rates in IC→LH connections (Fig. 3*F*) and low IC retrograde labelling in the LH (Fig. 9*C*) we limited further analyses to the mPFC→LH connections. LH→mPFC neurons had larger EPSP amplitudes in response to mPFC input compared to non‐mPFC projecting LH neurons (4.8 ± 4.5 mV *vs*. 2.2 ± 1.8 mV, *P* = 0.011, GLME, Fig. 9*G*) and shorter EPSP latency (4.6 ± 1.2 ms *vs*. 5.1 ± 1.2 ms, *P* = 0.016, GLME). We observed no differences in EPSP dynamics (EPSP5/EPSP1 ratios 1.3 ± 1.6 *vs*. 1.9 ± 3.0, *P* = 0.593, GLME). Overall although our data on the IC inputs are preliminary and limited by sample size, these data suggest that mPFC inputs are specialized to excite LH neurons which reciprocally project to the mPFC.

## Discussion

The cortex has a strong influence on motivated behaviours such as feeding (Baldo et al., 2016; Clarke et al., 2023; Hartmann et al., 2024; Mena et al., 2011; Mohammad et al., 2021; Stevenson & Francis, 2017; Uher & Treasure, 2005; Wu et al., 2020), known to be regulated by the LH (Anand & Brobeck, 1951; Jennings et al., 2015; Stamatakis et al., 2016). Our results suggest that these functions may be orchestrated through direct projections from L5/6 pyramidal neurons of frontotemporal association cortices to the LH. This adds to our current understanding of the synaptic drive of LH neurons, which was largely based on anatomical tracing studies that provide limited insight into synaptic strength, dynamics and integration. In addition previous mapping studies had typically been conducted in rats (Gabbott et al., 2005; Hahn & Swanson, 2012; Risold et al., 1997). The few anatomical studies in mice have reported inconsistent cortical labelling, particularly in IC, ECT and hippocampus (González et al., 2016; Hahn et al., 2022; Ugur et al., 2021). Anatomical connections from mPFC, IC and vSub have been electrophysiologically verified previously across unrelated studies (Mohammad et al., 2021; Padilla‐Coreano et al., 2022; Supiot et al., 2026; Tang et al., 2015; Wu et al., 2020). In this study we tested cortico‐LH connections under the same conditions, confirmed one new pathway from ECT and showed that the anatomically labelled pathway from dSub is functionally extremely weak and likely formed mostly of fibres of passage. This underscores the importance of our optogenetic assays, which added critical detail to our anatomical studies.

Our anatomical experiments showed a marked spatial continuity of L5/6 labelling across frontotemporal association cortices, suggesting that these projection neurons may form a more cohesive or interconnected network than previously appreciated. Such co‐ordination may be central to selective activation of individual LH neurons, as inputs from IC and mPFC were supralinearly integrated within individual LH neurons. Upstream mechanisms co‐ordinating LH inputs may be particularly critical as the LH itself lacks dense local circuitry (Burdakov & Karnani, 2020; Shao et al., 2022).

The supralinear integration of mPFC and IC inputs within individual LH neurons may stem from a variety of mechanisms, including voltage‐gated conductances and clustering of mPFC and IC synapses near each other. NMDA receptors are known to induce supralinear integration as they are gated by a coincidence of depolarization and glutamate release, and a clustering of mPFC and IC synapses on the same dendrites of LH neurons would ensure that the synaptic depolarization influences NMDA receptors (Branco et al., 2010).

mPFC and IC inputs to the LH displayed distinct synaptic dynamics and innervated partially non‐overlapping domains, suggesting functional specialization of these circuits. As thalamic outputs from L5 and L6 are known to exhibit depressing and facilitating dynamics, respectively (Collins et al., 2018; Zolnik et al., 2024), we speculate that the relative strengths of synapses from L5 *versus* L6 may underly the difference between mPFC and IC. Because our viral approach indiscriminately targeted projection neurons, we could not resolve whether differences in cortical layer or projection neuron subtype contribute to the observed synaptic properties.

Responsivity to mPFC *versus* IC input further correlated with electrophysiological characteristics (Fig. 8) of LH neurons. Dual‐channel optogenetics showed that IC‐preferring neurons had elevated excitability due to enhanced h‐currents. As this reduces the time window for synaptic integration (Shu et al., 2023), and IC→LH synapses are facilitating, these mechanisms may combine to allow IC‐preferring neurons to respond selectively to presynaptic burst firing. Moreover postinhibitory rebound excitability may make LH neurons particularly responsive after inhibitory inputs, for example, from the central amygdala (Weera et al., 2021). Anatomical and ultrastructural evidence supports colocalization of IC terminals with GABAergic central amygdala terminals in a dorsolateral LH region topographically similar to that identified in our anterograde experiments, though functional verification is still needed (Tsumori et al., 2006).

mPFC axons were more broadly distributed than IC axons and formed synapses with more LH neurons. Innervated LH neurons had mixed intrinsic characteristics consistent with multiple cell types, including melanin‐concentrating hormone, orexin, GABAergic and glutamatergic neurons (Karnani et al., 2013; Supiot et al., 2026). It is therefore likely that specialized ensembles of target‐specific mPFC→LH projection neurons are required to drive specific behaviours. This complicates interpretation of average activity recording data such as fibre photometry in the mPFC→LH population and may obscure distinct behavioural contributions in opto‐ or chemogenetic manipulation experiments. There was also marked variance in EPSP amplitude among mPFC recipient cells. Although some of this response variability may stem from technical factors, such as detection limitations in distal dendrites, synaptic strength is known to be actively regulated in LH neurons in response to caloric state (Horvath & Gao, 2005; Linehan et al., 2020), sleep/wake state (Rao et al., 2007), stress (Supiot et al., 2026) and cocaine exposure (Rao et al., 2013).

Our combined retrograde and anterograde experiment (Fig. 9) revealed that strongly mPFC‐recipient cells tended to project back to the mPFC, suggesting a direct feedback mechanism. Reciprocal connectivity is a common feature of PFC circuits, including its interactions with the thalamus (Anastasiades & Carter, 2021; Collins et al., 2018), which serve crucial functions in various cognitive processes by amplifying and sustaining representations in the PFC (Parnaudeau et al., 2018). In addition several studies underscore the importance of mPFC‐projecting LH neurons in learning cue‐food associations and cue‐driven consumption (Cole et al., 2020; Petrovich & Gallagher, 2007). Therefore the bidirectional communication between the mPFC and LH could help regulate adaptive behaviours and maintain cognitive control during complex tasks.

Overall these results identify the mPFC and IC as rapid cortical mediators of LH activity. The local integration of mPFC and IC inputs and the preferential reciprocal connectivity with the mPFC suggest that processing within individual neurons is critical in the LH. Further exploration of functional integration within individual LH neurons will be important for elucidating its operational principles across survival behaviours.

## Additional information

## Competing interests

The authors declare that they have no conflicts of interest.

## Author contributions

L.J.A.M.R.: conceptualization, methodology, formal analysis, investigation, visualization, supervision, writing – original draft. P.d.G.: data curation, investigation. H.D.M.: funding acquisition, supervision, writing – review and editing. M.M.K.: conceptualization, methodology, supervision, writing – review and editing. All authors have read and approved the final version of the manuscript and agreed to be accountable for all aspects of the work in ensuring that questions related to the accuracy or integrity of any part of the work are appropriately investigated and resolved. All persons designated as authors qualify for authorship, and all those who qualify for authorship are listed.

## Funding

This project was funded by the Dutch Research Council Gravitation project BRAINSCAPES: A Road map from Neurogenetics to Neurobiology, grant no.: 024.004.012. M.M.K. was funded by the University of Edinburgh Chancellor's Fellowship and Royal Society research grant RGS∖R2∖252414.

## Supporting information




Peer Review History

Statistical Summary

Supporting Information

## Data Availability

The data that support the findings of this study are available from the corresponding author upon reasonable request.
