# Supplementary material for: Integration of cortical inputs in the lateral hypothalamus is dominated by the medial prefrontal cortex
Source: J Physiol. 2026 Jun 16;604(14):6057–79. doi: 10.1113/JP288596 (PMC13370700; doi:10.1113/JP288596)
Supplement: Supplementary file 2 — Statistical Summary [file TJP-604-6057-s001.pdf]

Figure	Variable	Groups	n	Mean	SD	Test					p		Pairwise comparisons		Statistic		Adjusted-p
Fig 3																	
3F	Response rate	**mPFC**	49/79	62.03%		Chi Square		df	n	Chi-sq	p		Fisher's Exact tests		OddsRatio	p	fdr
		**aIC**	10/29	34.48%				3	201	21.38		**0.000**	mPFC	aIC	3.10	0.016	**0.032**
		**-IC-**	20/68	29.41%									mPFC	-IC-	3.92	0.000	**0.001**
		**pIC**	6/25	24.00%									mPFC	pIC	5.17	0.001	**0.003**
		ECT	2/20	10.00%									aIC	-IC-	1.26	0.638	0.765
		dSub	1/29	3.45%									aIC	pIC	1.67	0.552	0.765
		vSub	4/15	26.67%									-IC-	pIC	1.32	0.795	0.795
3G	Amplitude P1	**mPFC**	49	3.72 ±	3.81	Kruskal Wallis							Wilcoxon rank sum				
		**aIC**	10	0.81 ±	0.52	'Source'	'SS'	'df'	'MS'	'Chi-sq'	p		mPFC	aIC	1362.00	0.001	**0.004**
		**-IC-**	20	1.89 ±	3.52	'Groups'	8753.90	3	2918.00	16.62		**0.001**	mPFC	-IC-	1611.00	0.002	**0.007**
		**pIC**	6	2.15 ±	2.55	'Error'	32326.00	75	431.0147				mPFC	pIC	1168.00	0.174	0.349
		ECT	2	3.08 ±	1.54	'Total'	41080.00	78					aIC	-IC-	139.00	0.630	0.679
		dSub	1	5.11									aIC	pIC	80.00	0.635	0.679
		vSub	4	3.10 ±	4.30								-IC-	pIC	240.00	0.679	0.679
-	Max Amplitude	**mPFC**	49	4.26 ±	3.94	Kruskal Wallis							Wilcoxon rank sum				
		**aIC**	10	1.68 ±	1.12	'Source'	'SS'	'df'	'MS'	'Chi-sq'	p		mPFC	aIC	1317.00	0.018	0.091
		**-IC-**	20	2.72 ±	3.56	'Groups'	4839.50	3	1095.176	9.19		**0.027**	mPFC	-IC-	1553.00	0.030	0.091
		**pIC**	6	2.36 ±	2.40	'Error'	36241.00	75	483.2071				mPFC	pIC	1172.00	0.139	0.279
		ECT	2	4.51 ±	3.57	'Total'	41080.00	78					aIC	-IC-	139.00	0.630	0.945
		dSub	1	5.11									aIC	pIC	84.00	0.958	0.958
		vSub	4	4.49 ±	4.98								-IC-	pIC	249.00	0.924	0.958
3H	Latency	**mPFC**	49	5.63 ±	1.33	Kruskal Wallis							Wilcoxon rank sum				
		**aIC**	10	6.51 ±	1.35	'Source'	'SS'	'df'	'MS'	'Chi-sq'	p		mPFC	aIC	1092.00	0.119	0.239
		**-IC-**	20	6.41 ±	1.55	'Groups'	3688.60	3	1.23E+03	7.18		0.066	mPFC	-IC-	1244.00	0.093	0.239
		**pIC**	6	6.67 ±	0.39	'Error'	35851.00	74	484.4717				mPFC	pIC	1010.00	0.049	0.239
		ECT	2	5.22 ±	0.47	'Total'	39540.00	77					aIC	-IC-	158.00	0.731	0.731
		dSub	1	5.85									aIC	pIC	77.00	0.428	0.513
		vSub	4	4.93 ±	1.17								-IC-	pIC	226.00	0.192	0.288
3I	PPR	**mPFC**				GLME	PPR ~ Group * Pulsenr + (1|cellid)'										
		**aIC**				Term	Fstat	DF1	DF2		p						
		**-IC-**				Intercept	156.40	1	375			0.000					
		**pIC**				Group	0.24	3	375			0.869					
		ECT				PulseNr	0.36	4	375			0.836					
		dSub				Group * PulseNr	2.14	12	375			**0.014**					
		vSub															
3J	P5/P1	**mPFC**	49	0.89 ±	1.03	Kruskal Wallis							Wilcoxon rank sum		ranksum	p	fdr
		**aIC**	10	2.13 ±	1.75	'Source'	'SS'	'df'	'MS'	'Chi-sq'	p		mPFC	aIC	1062.00	0.001	**0.006**
		**-IC-**	20	1.98 ±	2.26	'Groups'	8635.13	3	2878.377	16.40		**0.001**	mPFC	-IC-	1212.00	0.003	**0.010**
		**pIC**	6	0.95 ±	0.94	'Error'	32444.87	75	432.5982				mPFC	pIC	1116.00	0.870	0.870
		ECT	2	0.85 ±	0.94	'Total'	41080.00	78					aIC	-IC-	176.00	0.242	0.290
		dSub	1	0.28 ±									aIC	pIC	103.00	0.056	0.112
		vSub	4	1.53 ±	0.45								-IC-	pIC	270.00	0.152	0.228

3K	Facilitation rate	**mPFC**	10/49	20.41%			Chi Square						Fisher's Exact tests		OddsRatio	p	fdr
		**aIC**	8/10	80.00%					df	n	Chi-sq	p	mPFC	aIC	0.06	0.001	**0.004**
		**-IC-**	9/20	45.00%					3	85	14.52	**0.002**	mPFC	-IC-	0.31	0.072	0.179
		**pIC**	2/6	33.33%									mPFC	pIC	0.51	0.602	0.722
		ECT	1/2	50.00%									aIC	-IC-	4.89	0.119	0.179
		dSub	0/1	0.00%									aIC	pIC	8.00	0.118	0.179
		vSub	3/4	75.00%									-IC-	pIC	1.64	1.000	1.000
2M	AP probability (per pulse)	**mPFC**					GLME	prob ~ group * Pulsenr + (1|cellID)									
		**aIC**					Term	Fstat	Df1	Df2		p					
		**-IC-**					Intercept	428.43	1	335		**0.000**					
		**pIC**					group	1.22	3	335		0.302					
		ECT					Pulsenr	1.81	4	335		0.126					
		dSub					group:Pulsenr	1.46	12	335		0.137					
		vSub															
	AP probability (1 or more AP)	**mPFC**	49	11.75%	±	0.04	Kruskal Wallis										
		**aIC**	10	0.00%	±	0.00	'Source'	'SS'	'df'	'MS'	'Chi-sq'	p					
		**-IC-**	20	1.85%	±	0.02	'Groups'	638.57	3	212.8555	4.10	0.251					
		**pIC**	6	6.67%	±	0.06	'Error'	10265.43	67	153.2154							
		ECT	2	0.00%	±	0.00	'Total'	10904.00	70								
		dSub	1	0.00%	±	0.00											
		vSub	4	0.00%	±	0.00											
3N	AP probabiliy vs. P5/P1	pooled	92				Linear Regression	R2				p					
								0.22				0.177					
	Monosynaptic confirmation																
	Amplitude	Baseline	19	3.94	±	4.77	Wilcoxon signed rank		n		zval	p					
		TTX+4-AP	19	6.39	±	6.78			19		-3.2427	**0.001**					
	Latency (ACSF)	Confirmed	19	5.79	±	1.29	Wilcoxon rank sum		n1	n1	zval	p					
		Unconfirmed	73	5.99	±	1.40			19	73	0.17	0.862					
Fig 5																	
	Mediolateral Peak Position of																
5E	KDE (0-100)	mPFC	3	63.75	±	7.29	GLME	var ~ gr*ap + (1|sample)'									
		IC	3	86.17	±	6.62	Term		Fstat	DF1	DF2	p					
							Intercept		843.4573	1	20.00	**0.000**					
							gr		25.7408	1	20.00	**0.000**					
							ap		1.2429	1	20.00	0.278					
							gr:ap		3.0883	1	20.00	0.094					
	Dorsoventral Peak Position of																
5F	KDE (0-100)	mPFC	3	23.42	±	7.29	GLME	var ~ gr*ap + (1|sample)'									
		IC	3	18.33	±	6.91	Term		Fstat	DF1	DF2	p					
							Intercept		66.755	1	20.00	**0.000**					

Fig	Measure	Group	n	Mean ± SD	Term					p						
					gr	5.3966		1	20.00	**0.031**						
					ap	1.1399		1	20.00	0.298						
					gr:ap	2.54		1	20.00	0.127						
5G	Total Area Fraction	mPFC	3	28% ± 7%	GLME	var ~ gr*ap + (1|sample)'										
		IC	3	12% ± 7%	Term	Fstat		DF1	DF2	p						
					Intercept	7.7405		1	20.00	**0.012**						
					gr	57.9087		1	20.00	**0.000**						
					ap	16.3309		1	20.00	**0.001**						
					gr:ap	23.72		1	20.00	**0.000**						
Fig 6																
6M	Desensitization	ChrimsonR$_{LED590}$	7	-41.92 ± 30.94	'Source'	'SS'	'df'	'MS'	'Chi-sq'	p	Wilcoxon Signed rank test			zval	p	FDR
		ChrimsonR$_{LED470}$	7	-31.15 ± 23.24	'Columns'	518.00	2	259	13.77	**0.001**	ChrimsonR	LED590	LED470	-1.521	0.128	0.128
		ChrimsonR$_{LED470\text{-}D}$	7	-0.08 ± 0.20	'Error'	234.50	18	13.02778			ChrimsonR	LED590	Desentizite	-2.366	0.018	**0.027**
					'Total'	752.50	20				ChrimsonR	LED470	Desentizite	-2.366	0.018	**0.027**
Fig 7																
7D	Desensitization	ChrimsonR$_{LED590}$	18	-28.82 ± 18.98							**Wilcoxon rank sum test**			Z	p	FDR
		ChrimsonR$_{LED470}$	18	-19.48 ± 13.51							ChrimsonR	Chronos	LED590	-2.5350	0.011	**0.034**
		ChrimsonR$_{LED590\text{-}D}$	18	0.00 ± 0.00							Chrimson	Both	LED590	-2.0281	0.043	0.092
		Chronos$_{LED590}$	5	0.00 ± 0.00							Chronos	Both	LED590	1.95	0.051	0.092
		Chonos$_{LED470}$	5	-15.55 ± 9.09							ChrimsonR	Chronos	LED470	-0.0722	0.942	0.942
		Chronos$_{LED470\text{-}D}$	5	-8.13 ± 2.12							Chrimson	Both	LED470	0.66	0.510	0.656
		Both$_{LED590}$	16	-37.63 ± 73.64							Chronos	Both	LED470	0.71	0.477	0.656
		Both$_{LED470}$	16	-35.39 ± 47.79							ChrimsonR	Chronos	Desens	3.61	0.000	**0.001**
		Both$_{LED470\text{-}D}$	16	-12.52 ± 9.83							Chrimson	Both	Desens	4.57	0.000	**0.000**
											Chronos	Both	Desens	0.12	0.906	0.942
	EPSC parameters				GLME	*var* ~ opsin*cortex + (1| cell)										
7E	Amplitude (pA)	mPFC$_{ChrimsonR}$	29	31.31 ± 54.34	Term	Fstat		DF1	DF2	p						
		mPFC$_{Chronos}$	15	13.23 ± 17.38	Intercept	4.7816		1	84.00	**0.032**						
		IC$_{ChrimsonR}$	29	4.11 ± 8.14	Cortex	3.9734		1	84.00	**0.050**						
		IC$_{Chronos}$	15	2.71 ± 5.50	Opsin	0.4493		1	84.00	0.505						
		mPFC pooled	44	25.15 ± 45.79	Cortex:Opsin	0.7879		1	84.00	0.377						
		IC pooled	44	3.63 ± 7.31												
7F	Latency (ms)	mPFC$_{ChrimsonR}$	29	3.89 ± 1.77	Intercept	91.4397		1	43.00	**0.000**						
		mPFC$_{Chronos}$	15	4.18 ± 1.85	Cortex	0.55		1	43.00	0.462						
		IC$_{ChrimsonR}$	29	5.31 ± 3.63	Opsin	1.947		1	43.00	0.170						
		IC$_{Chronos}$	15	4.85 ± 1.14	Cortex:Opsin	0.7719		1	43.00	0.385						

		mPFC pooled	44	3.99	±	1.77						
		IC pooled	44	5.19	±	3.16						
7G	Rise time (ms)	mPFC$_{ChrimsonR}$	29	2.58	±	1.61	Intercept	12.1211	1	41.00	**0.001**	
		mPFC$_{Chronos}$	15	2.00	±	1.27	Cortex	0.142	1	41.00	0.708	
		IC$_{ChrimsonR}$	29	3.57	±	3.72	Opsin	1.0281	1	41.00	0.317	
		IC$_{Chronos}$	15	2.37	±	0.78	Cortex:Opsin	2.2166	1	41.00	0.144	
		mPFC pooled	44	2.40	±	1.52						
		IC pooled	44	3.33	±	3.35						
7H	Decay time (ms)	mPFC$_{ChrimsonR}$	29	9.85	±	9.85	Intercept	28.481	1	41.00	**0.000**	
		mPFC$_{Chronos}$	15	4.61	±	4.61	Cortex	1.8519	1	41.00	0.181	
		IC$_{ChrimsonR}$	29	5.93	±	5.93	Opsin	0.0566	1	41.00	0.813	
		IC$_{Chronos}$	15	6.47	±	6.47	Cortex:Opsin	3.3657	1	41.00	0.074	
		mPFC pooled	44	8.20	±	7.07						
		IC pooled	44	6.03	±	4.07						
	Response integration						Wilcoxon signed rank test					
		**Integrating neurons**										
7K	Amplitude (pA)	Summed	10	69.73	±	90.40	Normalized to summed response	n	zval	p		
		Dual	10	97.18	±	45.65	Summed vs. Dual Stim		10	-2.0732	**0.038**	
		Dual_normalized	10	1.21	±	0.27						
7L	Surface area (pA * s)	Summed	10	0.57	±	0.69	Normalized to summed response		10	-2.0732	**0.038**	
		Dual	10	0.78	±	1.07	Summed vs. Dual Stim					
		Dual_normalized	10	1.26	±	0.35						
		**Chrimson Only Neurons**										
7N	Amplitude (pA)	Summed	9	32.70	±	20.79	Normalized to summed response	n	zval	p		
		Dual	9	30.93	±	20.19	Summed vs. Dual Stim		9	1.84	0.066	
		Dual_normalized	9	0.95	±	0.08						
7O	Surface area (pA * s)	Summed	9	0.30	±	0.21	Normalized to summed response					
		Dual	9	0.33	±	0.29	Summed vs. Dual Stim		9	-0.5601	0.575	
		Dual_normalized	9	1.04	±	0.22						
-	Amplitude (pA)	LED590 10ms	9	31.49	±	20.98	Normalized to red					
		Dual	9	30.93	±	20.19	Red light vs. Dual stim		9	-0.5331	0.594	
		Dual_normalized	9	1.01	±	0.09						
Fig 8												
8A	mPFC EPSC Amplitude	All	44	29.95	±	56.92						
		mPFC$_{Preferring}$	30	42.34	±	65.54						
		IC$_{Preferring}$	9	5.26	±	4.34						

	IC EPSC Amplitude	All	44	4.62 ± 7.60							
		$mPFC_{Preferring}$	30	2.26 ± 4.36							
		$IC_{Preferring}$	9	15.02 ± 9.28							
8B					Wilcoxon Signed rank tests						
	EPSC Amplitude				Normalized to max input			n	zval	p	
	$mPFC_{Preferring}$	mPFC EPSC	30	1.00 ± 0.00	mPFC vs. IC EPSC			30	4.99	**0.000**	
		IC EPSC	30	0.10 ± 0.22							
8D	EPSC Amplitude				Wilcoxon Signed rank tests						
	$IC_{Preferring}$	mPFC EPSC	9	0.41 ± 0.34	Normalized to max input			n	zval	p	
		IC EPSC	9	1.00 ± 0.00	mPFC vs. IC EPSC			9	-2.6679	**0.008**	
8G	Rinput	$mPFC_{Preferring}$	21	343.14 ± 159.46	Wilcoxon rank sum		n1	n2	zval	p	
		$IC_{Preferring}$	7	380.79 ± 119.94			21	7	-0.688	0.491	
-	Sag (mV)	$mPFC_{Preferring}$	21	4.45 ± 4.71	Wilcoxon rank sum		n1	n2	zval	p	
		$IC_{Preferring}$	7	11.68 ± 9.09			21	7	-2.1753	**0.033**	
-	Sag (ratio)	$mPFC_{Preferring}$	21	0.96 ± 0.03	Wilcoxon rank sum		n1	n2	zval	p	
		$IC_{Preferring}$	7	0.91 ± 0.06			21	7	2.07	**0.039**	
8H	Sag (% of peak deflection)	$mPFC_{Preferring}$	21	7.95 ± 6.90	Wilcoxon rank sum		n1	n2	zval	p	
		$IC_{Preferring}$	7	18.09 ± 11.96			21	7	-2.07	**0.039**	
8I	PIR	$mPFC_{Preferring}$	10/21	48%	Chi Square test		df	n	Chi-sq	p	
		$IC_{Preferring}$	6/7	86%			1	32	4.06	**0.044**	
	sEPSC analysis										
-	sEPSC frequency	$mPFC_{Preferring}$	19	5.74 ± 6.50	Wilcoxon rank sum		n1	n2	zval	p	
		$IC_{Preferring}$	8	5.08 ± 4.41			19	8	-0.3451	0.730	
-	sEPSC amplitude (abs)	$mPFC_{Preferring}$	19	30.82 ± 25.37	Wilcoxon rank sum		n1	n2	zval	p	
		$IC_{Preferring}$	8	22.24 ± 6.49			19	8	0.24	0.811	
	Current ramp										
-	Firing Rate	$mPFC_{Preferring}$	16		Repeated Measures ANOVA						
		$IC_{Preferring}$	5			SumSq	DF	MeanSq	F	pValue	
					(Intercept):Time	7715.69	24	321.4871	1.96	**0.005**	
					group:Time	5338.02	24	222.4175	1.36	0.122	
					Error(Time)	74732.99	456	163.8881	1.00	0.500	
-	Gain (Hz/pA)	$mPFC_{Preferring}$	16	0.32 ± 0.19	Wilcoxon rank sum		n1	n2	zval	p	
		$IC_{Preferring}$	5	0.30 ± 0.21			16	6	0.45	0.650	

Fig	Measure	Group	n	Mean ± SD	Test	Model				p
-	Rheobase	$mPFC_{Preferring}$	16	29.68 ± 27.99	Wilcoxon rank sum		n1	n2	zval	p
		$IC_{Preferring}$	5	44.35 ± 47.33			16	5	-0.7019	0.483
-	Max Firing rate	$mPFC_{Preferring}$	16	34.69 ± 18.27	Wilcoxon rank sum		n1	n2	zval	p
		$IC_{Preferring}$	5	35.33 ± 19.09			16	5	-0.0413	0.967
	Cell location				Wilcoxon rank sum		n1	n2	zval	p
8K	Medial-Lateral	$mPFC_{Preferring}$	30	0.33 ± 0.33			30	9	-1.9228	0.055
		$IC_{Preferring}$	9	0.58 ± 0.25						
8L	Dorsal-Ventral	$mPFC_{Preferring}$	30	0.44 ± 0.24			30	9	-2.3833	**0.017**
		$IC_{Preferring}$	9	0.63 ± 0.15						
8M	Anterior-Posterior	$mPFC_{Preferring}$	30	-1.38 ± 0.25			30	9	1.30	0.193
		$IC_{Preferring}$	9	-1.48 ± 0.22						
Fig 9										
9C	CTb counts	mPFC(total)	2	117.00	GLME	counts ~ Group * AP + (1 | sample)'				
	Total, across 9 slices	IC(total)	2	30.00	Term		Fstat	DF1	DF2	p
					Intercept		12.1414	1	32.00	**0.002**
					Group		11.2795	1	32.00	**0.002**
					AP		2.0197	1	32.00	0.165
					Group*AP		5.8567	1	32.00	**0.021**
9F	Response rate	$mPFC_{CTB+}$	11/11	100%	Chi Square test		df	n	Chi-sq	p
		$mPFC_{CTB-}$	10/15	67%	mPFC		1	26	4.54	**0.033**
		IC_{CTB+}	2/6	33%	IC		1	16	0.02	0.889
		IC_{CTB-}	3/10	30%						
9G	Max Ampl (mPFC)	$mPFC_{CTB+}$	11	4.80 ± 4.51	GLME	maxampl ~ gr + (1|rec) + (1|date:slice)'				
		$mPFC_{CTB-}$	16	2.19 ± 1.75	Term		Fstat	DF1	DF2	p
					Intercept		0.00	1	23.00	0.946
					Group		7.65	1	23.00	**0.011**
	Max Ampl (IC)	IC_{CTB+}	6	0.65 ± 0.86						
		IC_{CTB-}	10	1.10 ± 1.42						
-	Latency (mPFC)	$mPFC_{CTB+}$	11	4.64 ± 1.17	GLME	latency ~ gr + (1|rec) + (1|date:slice)'				
		$mPFC_{CTB-}$	10	5.10 ± 1.15	Term		Fstat	DF1	DF2	p
					Intercept		109.04	1	18.00	**0.000**
					Group		7.02	1	18.00	**0.016**
-	P5/P1 (mPFC)	$mPFC_{CTB+}$	11	1.32 ± 1.56	GLME	p5/p1 ~ gr + (1|rec) + (1|date:slice)'				
		$mPFC_{CTB-}$	10	1.85 ± 2.95	Term		Fstat	DF1	DF2	p
					Intercept		0.16	1	23.00	0.694
					Group		0.29	1	23.00	0.593

	ChrimsonR only neurons	590nm stimulation							
						Wilcoxon signed rank test	z	p	
	**Amplitude (pA)**	10ms	31	-32.71 ±	53.66		1.56	0.118	
		250ms	31	-41.92 ±	70.67				
	**Latency (ms)**	10ms	31	4.03 ±	1.56		1.37	0.171	
		250ms	31	3.44 ±	1.99				
	**Riste time(ms)**	10ms	31	2.53 ±	1.39		1.62	0.105	
		250ms	31	1.94 ±	1.28				
	**Decay time(ms)**	10ms	31	9.70 ±	8.18		0.88	0.379	
		250ms	31	6.84 ±	4.28				
