# Supplementary material for: Integration of cortical inputs in the lateral hypothalamus is dominated by the medial prefrontal cortex
Source: J Physiol. 2026 Jun 16;604(14):6057–79. doi: 10.1113/JP288596 (PMC13370700; doi:10.1113/JP288596)
Supplement: Supplementary file 1 — Peer Review History [file TJP-604-6057-s003.pdf]

The Journal of Physiology

Integration of cortical inputs in the lateral hypothalamus is dominated by the medial prefrontal cortex

Lotte Razenberg, Pieter Nicolaas de Greef, Huib Mansvelder, and Mahesh Miikael Karnani
DOI: 10.1113/JP288596

Corresponding author(s): Mahesh Karnani (mkarnani@ed.ac.uk)

The following individual(s) involved in review of this submission have agreed to reveal their identity: Ken D O'Halloran (Referee #3)

Review Timeline:

Submission Date:	22-Jan-2025
Editorial Decision:	11-Apr-2025
Revision Received:	08-Apr-2026
Editorial Decision:	01-May-2026
Revision Received:	03-May-2026
Accepted:	14-May-2026

Senior Editor: Nathan Schoppa

Reviewing Editor: Samuel Young

Transaction Report:

(Note: With the exception of the correction of typographical or spelling errors that could be a source of ambiguity, letters and reports are not edited. Depending on transfer agreements, referee reports obtained elsewhere may or may not be included in this compilation. Referee reports are anonymous unless the Referee chooses to sign their reports.)

Dear Dr Karnani,

Re: JP-RP-2025-288596 "**Integration of cortical inputs in the lateral hypothalamus is dominated by the medial prefrontal cortex**" by Lotte Razenberg, Pieter de Greef, Huib Mansvelder, and Mahesh Miikael Karnani

Thank you for submitting your manuscript to The Journal of Physiology. It has been assessed by a Reviewing Editor and by 2 expert referees and we are pleased to tell you that it is potentially acceptable for publication following satisfactory major revision.

Please advise your co-authors of this decision as soon as possible.

The referee reports are copied at the end of this email.

Please address all the points raised and incorporate all requested revisions or explain in your Response to Referees why a change has not been made. We hope you will find the comments helpful and that you will be able to return your revised manuscript within 9 months. If you require longer than this, please contact journal staff: jp@physoc.org. Please note that this letter does not constitute a guarantee for acceptance of your revised manuscript.

Your revised manuscript should be submitted online using the link in your Author Tasks: Link Not Available. This link is accessible via your account as Corresponding Author; it is not available to your co-authors. If this presents a problem, please contact journal staff (jp@physoc.org). Image files from the previous version are retained on the system. Please ensure you replace or remove any files that are being revised.

If you do not wish to submit a revised version of your manuscript, you must inform our journal staff (jp@physoc.org) or reply to this email to request withdrawal. Please note that a manuscript must be formally withdrawn from the peer review process at one journal before it may be submitted to another journal.

TRANSPARENT PEER REVIEW POLICY: To improve the transparency of its peer review process, The Journal of Physiology publishes online as supporting information the peer review history of all articles accepted for publication. Readers will have access to decision letters, including Editors' comments and referee reports, for each version of the manuscript, as well as any author responses to peer review comments. Referees can decide whether or not they wish to be named on the peer review history document.

ABSTRACT FIGURES: Authors are expected to use The Journal's premium BioRender account to create/redraw their Abstract Figures. Information on how to access this account is here: https://physoc.onlinelibrary.wiley.com/journal/14697793/biorender-access.

This will enable Authors to create and download high-resolution figures. If authors have used the free BioRender service, they can use the instructions provided in the link above to download a high-resolution version suitable for publication.

The link provided should only be used for the purposes of this submission. Authors will be charged for figures created on this account if they are not related to this manuscript submission.

LANGUAGE EDITING AND SUPPORT FOR PUBLICATION: If you would like help with English language editing, or other article preparation support, Wiley Editing Services offers expert help, including English Language Editing, as well as translation, manuscript formatting, and figure formatting at www.wileyauthors.com/eeo/preparation. You can also find resources for Preparing Your Article for general guidance about writing and preparing your manuscript at www.wileyauthors.com/eeo/prepresources.

RIGOUR AND REPRODUCIBILITY: We recommend authors consult The Journal's Rigour and Reproducibility requirements to ensure that their manuscript is suitable for publication: https://physoc.onlinelibrary.wiley.com/pb-assets/hub-assets/physoc/documents/TJP-Rigour-and-Reproducibility-Requirements.pdf

REVISION CHECKLIST:

Check that your Methods section conforms to journal policy: https://jp.msubmit.net/cgi-bin/main.plex?form_type=display_requirements#methods.

Check that data presented conforms to the statistics policy: https://jp.msubmit.net/cgi-bin/main.plex?form_type=display_requirements#statistics.

Upload a full Response to Referees file. To create your 'Response to Referees': copy all the reports, including any comments from the Senior and Reviewing Editors, into a Microsoft Word, or similar, file and respond to each point, using font or background colour to distinguish comments and responses and upload as the required file type.

Please upload two versions of your manuscript text: one with all relevant changes highlighted and one clean version with no

changes tracked. The manuscript file should include all tables and figure legends, but each figure/graph should be uploaded as separate, high-resolution files.

You may also upload:

- 'Potential Cover Art' for consideration as the issue's cover image
- Appropriate Supporting Information (Video, audio or data set: see https://jp.msubmit.net/cgi-bin/main.plex?form_type=display_requirements#supp).

We look forward to receiving your revised submission.

If you have any queries, please reply to this email and we will be pleased to advise.

Yours sincerely,

Katalin Toth
Senior Editor
The Journal of Physiology

REQUIRED ITEMS

- Author photo and profile. First or joint first authors are asked to provide a short biography (no more than 100 words for one author or 150 words in total for joint first authors) and a portrait photograph. These should be uploaded and clearly labelled together in a Word document with the revised version of the manuscript. See Information for Authors for further details.

- You must start the Methods section with a paragraph headed Ethical approval (https://jp.msubmit.net/cgi-bin/main.plex?form_type=display_requirements#methods).

Research must comply with The Journal's policies regarding animal experiments (https://physoc.onlinelibrary.wiley.com/hub/animal-experiments) and adherence to these policies must be stated in the manuscript.

Authors should confirm in their Methods section that their experiments were carried out according to the guidelines laid down by their institution's animal welfare committee, including an ethics approval reference number. The Methods section must contain a statement about access to food, water and housing, details of the anaesthetic regime: anaesthetic used, dose and route of administration, and method of killing the experimental animals.

- The reference list must be in alphabetical order, rather than numbered, to comply with our Journal format.

- Please upload separate high-quality figure files via the submission form.

- Please ensure that the Article File you upload is a Word file.

- Your paper contains Supporting Information of a type that we no longer publish, including supplementary tables and figures. Any information essential to an understanding of the paper must be included as part of the main manuscript and figures. The only Supporting Information that we publish are video and audio, 3D structures, program codes and large data files. Your revised paper will be returned to you if it does not adhere to our Supporting Information Guidelines.

- Papers must comply with the Statistics Policy: https://jp.msubmit.net/cgi-bin/main.plex?form_type=display_requirements#statistics.

In summary:

- If n {less than or equal to} 30, all data points must be plotted in the figure in a way that reveals their range and distribution. A bar graph with data points overlaid, a box and whisker plot or a violin plot (preferably with data points included) are acceptable formats.

- If n > 30, then the entire raw dataset must be made available either as supporting information, or hosted on a not-for-profit repository, e.g. FigShare, with access details provided in the manuscript.

- 'n' clearly defined (e.g. x cells from y slices in z animals) in the Methods. Authors should be mindful of pseudoreplication.

- All relevant 'n' values must be clearly stated in the main text, figures and tables.

- The most appropriate summary statistic (e.g. mean or median and standard deviation) must be used. Standard Error of the Mean (SEM) alone is not permitted.

- Exact p values must be stated. Authors must not use 'greater than' or 'less than'. Exact p values must be stated to three significant figures even when 'no statistical significance' is claimed.

- Please include an Abstract Figure file, as well as the Figure Legend text within the main article file. The Abstract Figure is a piece of artwork designed to give readers an immediate understanding of the research and should summarise the main conclusions. If possible, the image should be easily 'readable' from left to right or top to bottom. It should show the physiological relevance of the manuscript so readers can assess the importance and content of its findings. Abstract Figures should not merely recapitulate other figures in the manuscript. Please try to keep the diagram as simple as possible and without superfluous information that may distract from the main conclusion(s). Abstract Figures must be provided by authors no later than the revised manuscript stage and should be uploaded as a separate file during online submission labelled as File Type 'Abstract Figure'. Please also ensure that you include the figure legend in the main article file. All Abstract Figures should be created using BioRender. Authors should use The Journal's premium BioRender account to export high-resolution images. Details on how to use and access the premium account are included as part of this email.

EDITOR COMMENTS

Reviewing Editor:

Comments for Authors to ensure the paper complies with the Statistics Policy (Required):
The data must be reported as SD not SEM

Comments to the Author (Required):
This manuscript examines the functional properties of cortical inputs in the lateral hypothalamus. Both reviewers found the data interesting and the study to be impactful. However, both reviewers have concerns about the manuscript in particular conclusions drawn based on the current experiments were not supported and that conclusions were overstated. Therefore, they will need to be addressed by additional experiments and significant text revisions. To strengthen conclusions in the papers, the authors will need to perform additional control experiments that clearly demonstrate that out form Chrimson R-expressing neurons remain unchanged. They will need to increase the number of animals used for experiments involving reciprocally connected neurons to draw a meaningful conclusion. Both reviewers noted that the text in current state was difficult to read and lacked significant explanation of experiments and how analysis was performed. Therefore, based on these positive comments and critiques a significant revision of the text will need to be done. Furthermore, the manuscript will need to comply with Journal policies for reporting of statistics as SD and manuscript formatting.

Please also see 'Required Items' above.

REFEREE COMMENTS

Referee #1:

In this manuscript, Razenberg et al. utilize in vitro electrophysiology, optogenetics, and anatomical tracing to explore the functional properties of cortical inputs on neurons in the lateral hypothalamus (LH). The LH plays a crucial role in regulating

various physiological and behavioral functions, making it essential to understand how different cortical inputs influence LH activity. Through a series of experiments, the authors identify the medial prefrontal cortex (mPFC) and the insular cortex (IC) as two significant sources of cortical input to the LH. They observe differences in the projection patterns and functional properties of the synapses from these two regions. By employing dual-optogenetics strategies, they report that about one-third of LH neurons receive input from both the mPFC and IC, integrating these inputs in a supra-linear manner. Furthermore, the authors find that LH neurons with strong IC inputs are electrophysiologically distinct. They also note that mPFC projections, but not those from the IC, preferentially target LH neurons that are reciprocally connected.

The manuscript is generally well-written and well-illustrated, but there are areas for improvement. Most of the experiments were thoughtfully designed and executed; however, some of the conclusions are unconvincing based on what is presented. Although the data is well analyzed, there are missing descriptions for some data. Additionally, the introduction is too short, focusing heavily on the results of the paper without adequately establishing the significance of the work. Other minor weaknesses dampened overall excitement for this version of the manuscript. Below are my specific comments:

Major Concerns

1. The dual integration test fails to provide convincing evidence. The authors must clarify how they can rule out the possibility that their observed effects stem from increased excitability in the ChrimsonR-expressing axons/terminals when stimulated simultaneously with both red and blue light. To strengthen their conclusions, it is essential to conduct a control experiment that clearly demonstrates that the output from ChrimsonR-expressing cortical axons remains unchanged when comparing red light stimulation to simultaneous stimulation with both red and blue lights. This additional evidence would significantly enhance their findings.

2. The observed difference in sag potential is questionable. There is substantial variability in the amount of sag present between the two populations, which raises concerns. While the authors state that voltage sag was measured with hyperpolarizing steps, they fail to provide essential details. What was the specific step size used? How did they account for cell-specific differences in input resistance? This information is critical, as the magnitude of the sag is likely influenced by the initial peak potential. To enhance the rigor of their findings, the authors must clearly articulate how their data were quantified and how they controlled for variations in input resistance.

3. Our observations indicate that repetitive prolonged 250 ms light stimulation significantly alters the release probability of ChrimsonR-expressing axons. Was there any evidence that the amplitude of the 10 ms red light responses changed across these repetitive trials? If so, what measures were taken to control this?

4. The description of how short-term dynamics were measured is lacking details. In the methods section, the authors state they measured the ratio by dividing the amplitude of each pulse by the amplitude of the first light pulse in the train. However, many LH cells exhibit significant summation of optically evoked EPSPs, while others do not (see Figure 2, 5 and S2). How did the authors account for this summation in their measurements? Providing clarity on this is important for understanding their findings. Relating the different EPSP kinetics to the sag/Ih difference highlighted in the manuscript (Related to point 2 above) could also provide an interesting discussion point about how LH neurons integrate cortical inputs.

5. The introduction is notably short, with almost half of it focused on presenting the results. To elevate the manuscript's significance and broaden its appeal, the authors should more effectively highlight the importance of the problem and address the critical gaps in knowledge that this research aims to fill.

Minor

6. The authors must clarify whether retrogradely labeled neurons were recorded simultaneously with non-labeled cells, or

sequential. If sequential, were positive and negative cells adjacent? If positive and negative cells were not recorded simultaneously or sequentially, it is crucial to explain how differences in opsin expression were controlled for across cells, slices, and preparations. Clarification of this information is essential for understanding the validity of the results.

7. The authors used AAV1 in their experiments, yet it is well-established that AAV1 possesses significant anterograde transsynaptic spread capabilities. Did the authors detect any evidence of anterograde expression of opsins in the recorded cells, even if it was only subtle? Commenting on this issue in the manuscript is important.

8. The LH comprises several neuronal subtypes, including excitatory and inhibitory neurons, and yet this is not mentioned in the manuscript. A better discussion of these various cell types and their potential relationships to the observed results would greatly improve the manuscript.

9. For the dual-opsin experiments, the methods state that amplitude comparisons were done by comparing the 10 ms light pulse responses, which is the correct approach for analyzing these data. However, in Figures 4B-D, it appears that a comparison is being made between the 590 nm, 250 ms light pulse and the 470 nm, 10 ms light pulse. This raises a critical question: Were these data analyzed differently from those presented in Figure 3? If so, such comparisons could undermine the results. If not, it is critical to revise the presentation of these data to prevent confusion. Clearer communication and presentation are important.

10. In multiple brain regions, it has been established that excitatory cells in layers 5 (L5) and 6 (L6) display distinct synaptic properties. Could the authors provide insights on whether the short-term dynamics observed are influenced by the ratio of L5 to L6 cells labeled in these specific brain regions? Adding a discussion point on this topic would also be helpful.

11. Figure 2 emphasizes the modest short-term depression of mPFC inputs to LH neurons (Figure 2 G), and yet the AP probability increases with spike number. These two pieces of data don't necessarily match. Can the authors elaborate on these data? Is there enough evidence to suggest there are two types of mPFC synaptic responses (facilitating and depressing)? For example, the progressive pulse ratio plots in Figure S2 seem to highlight two types of responses for mPFC and perhaps others. This could relate to the fact the authors observe both L5 and L6 labeled (related to point 10 above).

12. The horizontal lines above Figure 4 F and J seem unnecessary

13. The titers of the viruses used is not reported; despite saying it is in Table 1

14. What was the N for the mPFC data in Figures S4.

15. Figure 5G says 11 of 11 mPFC cells, but the text in the results says 10/10 cells.

16. Discussion, paragraph 3: the term "powerful" is overstated given the overall weak nature of the inputs (<5 mV) and low probability of driving spiking activity in LH cells.

17. Figure 4 legend: The description for panel A and C refers to seeing figure 4. I think the authors mean figure 3.

18. Scales are missing values or are not reported in the legend for several figures.

19. Testing the dual-opsin interactions over cell bodies is not the same as testing over axons/terminals and it is unclear why these experiments were done and what they add.

20. The references were not displayed throughput the manuscript, making it challenging and annoying to check references.

Referee #2:

Ms # JP-RP-2025-288596

Integration of cortical inputs in the lateral hypothalamus is dominated by the medial prefrontal cortex

by Razenberg, de Greef, Mansvelder, Karnani

In this study the authors employed anatomical tracing and optogenetic circuit mapping in mice to identify and characterise distinct excitatory cortical (including subicluar and hippocampal) inputs to the lateral hypothalamus (LH), with a particular focus on the medial prefrontal cortex (mPFC) and the insular cortex (IC). The authors show that the mPFC provides the most widespread and potent monosynaptic input, apparently followed by the IC, subiculum, and ectorhinal cortex. The study revealed that insular inputs preferentially target a highly excitable dorsolateral subpopulation of LH neurons, characterised by a pronounced h-current (Ih) and post-inhibitory rebound. In contrast, mPFC inputs extensively innervate LH neurons that project back to the mPFC, i.e reciprocally connected LH neurons. LH neurons that convergent input from mPFC and IC, simultaneous stimulation sums supralinearly, suggesting robust potential for cortical co-activation driving powerful LH output. The authors propose that these findings reveal a specialised, pathway-specific integration within the LH that supports rapid, reciprocal communication with higher-order cortical regions involved in survival behaviours.

However, while I consider this study to be very interesting, at least the writing and editing of the manuscript leaves much to be desired and is not acceptable in its present form. It is very obvious that the manuscript was initially intend for a different journal because the authors did not even make the effort to change the format to J. Physiol. style (i.e. Abstract, Introduction, Methods, Results, Discussion). Moreover, and very annoyingly so, the citation style was not altered and but not even checked because upon submission the in-text citations became incomplete so that it is difficult to refer back to the actual paper(s) cited (see my comments below).

Furthermore, it is not entirely clear which findings of the study shown for the first time in this work and are not only supporting earlier studies. In particular, the retrograde tracing studies performed in this study have previously been done using other tracers and led to very similar conclusions. Here it would have been nice to seen how cholera toxin b (CTb) labels individual axons or presynaptic structures. This would have greatly enhanced the findings of the study.

Specific points

In the main text body and figure legends, abbreviations are used that have not been introduced previously. Several figure legends appear incomplete or insufficiently detailed. In every legend, clarify the abbreviations, the sample sizes, and whether error bars represent SEM or SD. Provide sufficient information for readers to interpret each panel without referring back to the main text.

Citation style

It appears that this manuscript was previously submitted to another journal which uses a different citation style. When converting the manuscript for submission to the Journal of Physiology, the citation style was not checked and converted, resulting in citations that are not fully legible and/or incomplete. Please convert the current numerical inline citations to a style that conforms to the conventions of this journal (e.g. 'Smith et al., 2020'). Ensure that all in-text citations correctly match the references in the reference list and that references are fully legible and consistently formatted. Furthermore, citations should use unified journal names (e.g. either Current Biology or Curr Biol but not both).

Statistical Reporting

The rationale for using SEM instead of SD should be clarified, especially where sample sizes are small (e.g., n=4). SEM can underestimate the variability in small samples, so be sure to justify why SEM is most appropriate and how many independent animals versus slices or cells were used.

Displaying error bars for a dataset with only two data points (as e.g. in Fig. 2) is not acceptable and any statistical comparison is unlikely to be rigorous. I suggest to simply describe the results qualitatively

For all data shown here, please discuss whether such a disparity in sample size affects the reliability of any statistical test. For example, when describing synaptic dynamics you compare in Figure 2H,I data from mPFC (e.g., 11 of 49 cells) and the ventral subiculum (3 of 4 cells), i.e. with a sample size that differs by a factor of more than 10. If you retain those analyses, justify the choice and interpret with caution.

Results

The authors use a lot of expressions such as Latencyunconfirmed and Latencyconfirmed, Z, U, p, adj-p, $\chi 2$ (and several others) without explaining their meaning. This should be amended.

Page 3, 5th para.

Should read either 'fibre of passage' or 'en passant fibres'.

Page 4, 5th para. and Figure 2G-I

The authors state that 'Responses to these inputs displayed distinct synaptic dynamics (Fig. 2G). The paired-pulse ratio (EPSP5/EPSP1) was higher for IC and aIC inputs compared to mPFC inputs, indicating facilitation of responses to insular inputs'. However, when comparing the EPSP2/EPSP1 paired pulse ratio (PPR) it appears that the ventral subiculum response has a stronger facilitation than the aIC; in addition the pIC has a substantially lower PPR than vSub. Please clarify.

In this context, please describe the rationale for using the EPSP5/EPSP1 rather than the EPSP2/EPSP1 ratio.

As shown in Fig. 2H,I, the sample size for the pIC, the vSub and in particular the ECT and dSub is rather low and is likely to result in an over-interpretation of data. Please consider excluding or at least separating such minimal data.

Please clarify the meaning of '100% facilitation' for the y-axis label of Figure 2I. I assume this is supposed to mean 100% of all responses show facilitation but this axis label is misleading. Please rephrase.

Page 4, 5th para. and Figure 2K,L: Action Potential (AP) Probability

The authors state that 'Action potential (AP) firing was only observed in response to mPFC and IC stimulation (Fig. 2J-L)'. However, no AP firing was observed for the aIC and the -IC- and pIC showed a response in one case each (panel L). For all other input regions (ECT, dSub and vSub) the sample size is limited. Please provide a statistical comparison for the data to make firm conclusions.

Figure 3B Legend

Update this legend to include a complete description of panels, labelling, sample sizes, and any color coding or arrows that appear in the figure. Indicate precisely which cortical injection sites or transgenes each panel corresponds to.

Page 7, paragraphs 1-3

To these for preferential targeting of identified reciprocally connected LH neurons by mPFC and IC afferents, the authors used co-injections of Ctb and channelrhodopsin 2 (ChR2). For this, two mice for each neocortical region were used. This is quite low in particular in the light of the statement that the data suggest that 'mPFC inputs are specialised to excite reciprocally connected LH neurons'. Please indicate also the fraction of reciprocally connected LH neurons in mPFC and IC.

Figure 5

In panel C, the x-axis label (AP position) should be clarified. In addition, the y-axis label 'CTb counts' is somewhat misleading and probably means no. of CTb-positive neurons. Please change.

In panel E, please clarify what the example traces show.

Discussion

Labelling Data

While the retrograde tracing results are consistent with prior studies (e.g., Hosoya & Matsushita, 1980; Kita & Oomura, 1982), the manuscript should more explicitly highlight what is novel in these new experiments. Emphasise how your findings on subregional or cell-type-specific targeting go beyond older data. Otherwise, the impression is that the labelling results replicate mostly 'known' pathways.

Description of Supralinearity

It would be helpful to explain whether the observed supralinear integration depends on particular stimulation intensities or frequencies. Are there parameters that prevent a supralinear summation when mPFC and IC inputs are co-activated? If the data suggest that even modest co-activation triggers a supralinear response, clarify this explicitly.

Provide a more detailed explanation of how the 'sum versus dual stimulation' ratio was computed, and note any conditions under which that ratio might not exceed 1.0.

In the discussion, it would be helpful if you could suggest some potential mechanisms for the supralinear summation of mPFC and IC inputs and describe them in sufficient detail as this is an important and novel finding.

Page 8, 4th para.

You mention specifically that '... post-inhibitory rebound makes these neurons particularly responsive after subcortical inhibition, possibly from topographically colocalized inputs from the central amygdala, which could modulate their activity depending on timing ...'. This is an interesting point. However, I suggest to briefly explain the mPFC-LH-CA neuronal circuitry and the evidence is for macro- and/or microscopic colocalisation of these inputs.

Should either read Ih of h current or HCN channel current.

Page 8, 5th para.

What is meant by 'canonical' LH neuron subtypes? I

Abbreviations such as MCH and Orx neurons for Melanin-concentrating hormone and Orexin neurons, respectively should be introduced or not used at all if they appear only once.

Consistency Checks and Inconsistencies

Please review the Methods, Figure legends, and main text to ensure consistent reporting of sample sizes (n for cells, slices, animals), experimental groups, and statistical thresholds. Inconsistencies in reported group numbers or p-values can confuse readers.

END OF COMMENTS

In this manuscript, Razenberg et al. utilize in vitro electrophysiology, optogenetics, and anatomical tracing to explore the functional properties of cortical inputs on neurons in the lateral hypothalamus (LH). The LH plays a crucial role in regulating various physiological and behavioral functions, making it essential to understand how different cortical inputs influence LH activity. Through a series of experiments, the authors identify the medial prefrontal cortex (mPFC) and the insular cortex (IC) as two significant sources of cortical input to the LH. They observe differences in the projection patterns and functional properties of the synapses from these two regions. By employing dual-optogenetics strategies, they report that about one-third of LH neurons receive input from both the mPFC and IC, integrating these inputs in a supra-linear manner. Furthermore, the authors find that LH neurons with strong IC inputs are electrophysiologically distinct. They also note that mPFC projections, but not those from the IC, preferentially target LH neurons that are reciprocally connected.

The manuscript is generally well-written and well-illustrated, but there are areas for improvement. Most of the experiments were thoughtfully designed and executed; however, some of the conclusions are unconvincing based on what is presented. Although the data is well analyzed, there are missing descriptions for some data. Additionally, the introduction is too short, focusing heavily on the results of the paper without adequately establishing the significance of the work. Other minor weaknesses dampened overall excitement for this version of the manuscript. Below are my specific comments:

Major Concerns

1. The dual integration test fails to provide convincing evidence. The authors must clarify how they can rule out the possibility that their observed effects stem from increased excitability in the ChrimsonR-expressing axons/terminals when stimulated simultaneously with both red and blue light. To strengthen their conclusions, it is essential to conduct a control experiment that clearly demonstrates that the output from ChrimsonR-expressing cortical axons remains unchanged when comparing red light stimulation to simultaneous stimulation with both red and blue lights. This additional evidence would significantly enhance their findings.

2. The observed difference in sag potential is questionable. There is substantial variability in the amount of sag present between the two populations, which raises concerns. While the authors state that voltage sag was measured with hyperpolarizing steps, they fail to provide essential details. What was the specific step size used? How did they account for cell-specific differences in input resistance? This information is critical, as the magnitude of the sag is likely influenced by the initial peak potential. To enhance the rigor of their findings, the authors must clearly articulate how their data were quantified and how they controlled for variations in input resistance.

3. Our observations indicate that repetitive prolonged 250 ms light stimulation significantly alters the release probability of ChrimsonR-expressing axons. Was there any evidence that the amplitude of the 10 ms red light responses changed across these repetitive trials? If so, what measures were taken to control this?

4. The description of how short-term dynamics were measured is lacking details. In the methods section, the authors state they measured the ratio by dividing the amplitude of each pulse by the amplitude of the first light pulse in the train. However, many LH cells exhibit significant summation of optically evoked EPSPs, while others do not (see Figure 2, 5 and S2). How did the authors account for this summation in their measurements? Providing clarity on this is important for understanding their findings. Relating the different EPSP kinetics to the sag/Ih difference highlighted in the manuscript (Related to point 2 above) could also provide an interesting discussion point about how LH neurons integrate cortical inputs.

5. The introduction is notably short, with almost half of it focused on presenting the results. To elevate the manuscript's significance and broaden its appeal, the authors should more effectively highlight the importance of the problem and address the critical gaps in knowledge that this research aims to fill.

Minor

6. The authors must clarify whether retrogradely labeled neurons were recorded simultaneously with non-labeled cells, or sequential. If sequential, were positive and negative cells adjacent? If positive and negative cells were not recorded simultaneously or sequentially, it is crucial to explain how differences in opsin expression were controlled for across cells, slices, and preparations. Clarification of this information is essential for understanding the validity of the results.

7. The authors used AAV1 in their experiments, yet it is well-established that AAV1 possesses significant anterograde transsynaptic spread capabilities. Did the authors detect any evidence of anterograde expression of opsins in the recorded cells, even if it was only subtle? Commenting on this issue in the manuscript is important.

8. The LH comprises several neuronal subtypes, including excitatory and inhibitory neurons, and yet this is not mentioned in the manuscript. A better discussion of these various cell types and their potential relationships to the observed results would greatly improve the manuscript.

9. For the dual-opsin experiments, the methods state that amplitude comparisons were done by comparing the 10 ms light pulse responses, which is the correct approach for analyzing these data. However, in Figures 4B-D, it appears that a comparison is being made between the 590 nm, 250 ms light pulse and the 470 nm, 10 ms light pulse. This raises a critical question: Were these data analyzed differently from those presented in Figure 3? If so, such comparisons could

undermine the results. If not, it is critical to revise the presentation of these data to prevent confusion. Clearer communication and presentation are important.

10. In multiple brain regions, it has been established that excitatory cells in layers 5 (L5) and 6 (L6) display distinct synaptic properties. Could the authors provide insights on whether the short-term dynamics observed are influenced by the ratio of L5 to L6 cells labeled in these specific brain regions? Adding a discussion point on this topic would also be helpful.

11. Figure 2 emphasizes the modest short-term depression of mPFC inputs to LH neurons (Figure 2 G), and yet the AP probability increases with spike number. These two pieces of data don't necessarily match. Can the authors elaborate on these data? Is there enough evidence to suggest there are two types of mPFC synaptic responses (facilitating and depressing)? For example, the progressive pulse ratio plots in Figure S2 seem to highlight two types of responses for mPFC and perhaps others. This could relate to the fact the authors observe both L5 and L6 labeled (related to point 10 above).

12. The horizontal lines above Figure 4 F and J seem unnecessary

13. The titers of the viruses used is not reported; despite saying it is in Table 1

14. What was the N for the mPFC data in Figures S4.

15. Figure 5G says 11 of 11 mPFC cells, but the text in the results says 10/10 cells.

16. Discussion, paragraph 3: the term "powerful" is overstated given the overall weak nature of the inputs (<5 mV) and low probability of driving spiking activity in LH cells.

17. Figure 4 legend: The description for panel A and C refers to seeing figure 4. I think the authors mean figure 3.

18. Scales are missing values or are not reported in the legend for several figures.

19. Testing the dual-opsin interactions over cell bodies is not the same as testing over axons/terminals and it is unclear why these experiments were done and what they add.

20. The references were not displayed throughput the manuscript, making it challenging and annoying to check references.

We thank the Reviewing Editor and Referees for their thoughtful and constructive feedback, and have revised the manuscript accordingly. We include our responses to the comments below in green.

EDITOR COMMENTS

Reviewing Editor:

The data must be reported as SD not SEM
SD now used throughout.

This manuscript examines the functional properties of cortical inputs in the lateral hypothalamus. Both reviewers found the data interesting and the study to be impactful. However, both reviewers have concerns about the manuscript in particular conclusions drawn based on the current experiments were not supported and that conclusions were overstated. Therefore, they will need to be addressed by additional experiments and significant text revisions.
We appreciate the reviewers' thoughtful and constructive feedback, as well as their recognition of the interest and potential impact of our study. We understand the concerns regarding the strength of the conclusions drawn from the current dataset and have undertaken a thorough revision of the manuscript, tempering our conclusions and adding several new analyses and figure panels. We believe these changes have strengthened the manuscript and brought the conclusions in line with the data presented.

To strengthen conclusions in the papers, the authors will need to perform additional control experiments that clearly demonstrate that out form Chrimson R-expressing neurons remain unchanged.
We have now added a comparison of neurons responding only to ChrimsonR input and show that the amplitude of the responses to red light equals that of combined red and blue light. This finding is in line with our control experiments, where we show that EPSC amplitudes are saturated with our stimulation parameters and additional power from the blue light source should have no additional effect on response amplitude.

They will need to increase the number of animals used for experiments involving reciprocally connected neurons to draw a meaningful conclusion.
We have clarified the number of animals used for each experimental condition. Specifically, for the electrophysiological experiments, we used 6 animals for mPFC injections and 3 animals for IC injections, resulting in recordings from 27 and 16 LH neurons, respectively. We aplogise that these numbers were not clearly communicated in the original version, and they are now included in both the Methods and Results sections. For IC, the number of CTb+ LH neurons was lower (Fig. 9C), as was the likelihood of recordings with a synaptic response (Fig. 3F). Therefore, due to the limited sample size, we do not perform statistical analyses for the IC group and have rephrased our conclusions more cautiously, focusing on the preferential targeting of reciprocally connected LH neurons by mPFC inputs, and noting that data from the IC group are preliminary and limited by sample size.

Both reviewers noted that the text in current state was difficult to read and lacked significant explanation of experiments and how analysis was performed. Therefore, based on these positive comments and critiques a significant revision of the text will need to be done.

We have now made significant revisions of the text to improve readability.

Furthermore, the manuscript will need to comply with Journal policies for reporting of statistics as SD and manuscript formatting.
We have now edited accordingly.

Please also see 'Required Items' above.
We have now added the missing items.

REFEREE COMMENTS
Referee #1:

In this manuscript, Razenberg et al. utilize in vitro electrophysiology, optogenetics, and anatomical tracing to explore the functional properties of cortical inputs on neurons in the lateral hypothalamus (LH). The LH plays a crucial role in regulating various physiological and behavioral functions, making it essential to understand how different cortical inputs influence LH activity. Through a series of experiments, the authors identify the medial prefrontal cortex (mPFC) and the insular cortex (IC) as two significant sources of cortical input to the LH. They observe differences in the projection patterns and functional properties of the synapses from these two regions. By employing dual-optogenetics strategies, they report that about one-third of LH neurons receive input from both the mPFC and IC, integrating these inputs in a supra-linear manner. Furthermore, the authors find that LH neurons with strong IC inputs are electrophysiologically distinct. They also note that mPFC projections, but not those from the IC, preferentially target LH neurons that are reciprocally connected.

The manuscript is generally well-written and well-illustrated, but there are areas for improvement. Most of the experiments were thoughtfully designed and executed; however, some of the conclusions are unconvincing based on what is presented. Although the data is well analyzed, there are missing descriptions for some data. Additionally, the introduction is too short, focusing heavily on the results of the paper without adequately establishing the significance of the work.
We have now re-written the introduction to articulate the significance of our work, and added clearer descriptions of the data.

Other minor weaknesses dampened overall excitement for this version of the manuscript. Below are my specific comments:

Major Concerns
1. The dual integration test fails to provide convincing evidence. The authors must clarify how they can rule out the possibility that their observed effects stem from increased excitability in the ChrimsonR-expressing axons/terminals when stimulated simultaneously with both red and blue light. To strengthen their conclusions, it is essential to conduct a control experiment that clearly demonstrates that the output from ChrimsonR-expressing cortical axons remains unchanged when comparing red light stimulation to simultaneous stimulation with both red and blue lights. This additional evidence would significantly enhance their findings.

Thank you for raising this important concern. We conducted an additional analysis on LH neurons receiving input exclusively from ChrimsonR-expressing cortical axons. As shown in the new Fig. 7M-O, these cells did not exhibit supralinear summation. Their responses to red light stimulation were indistinguishable from their responses to dual red and blue light (see fig below). This finding is consistent with our earlier control experiments demonstrating that EPSC amplitudes are saturated at the applied stimulation intensities (Fig. 6), such that additional light energy from the blue LED would not be expected to further increase synaptic output. We have updated the Results and Discussion sections accordingly.

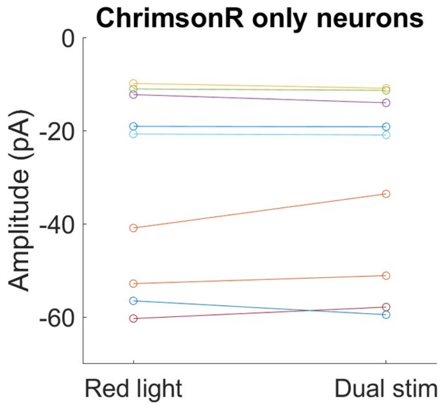

2. The observed difference in sag potential is questionable. There is substantial variability in the amount of sag present between the two populations, which raises concerns. While the authors state that voltage sag was measured with hyperpolarizing steps, they fail to provide essential details. What was the specific step size used? How did they account for cell-specific differences in input resistance? This information is critical, as the magnitude of the sag is likely influenced by the initial peak potential. To enhance the rigor of their findings, the authors must clearly articulate how their data were quantified and how they controlled for variations in input resistance.

These methodological clarifications have now been incorporated into the Methods section and referenced in the Results: Sag potential was calculated as the difference between the peak and steady-state membrane potential during a –150 pA hyperpolarizing step. To account for cell-to-cell variability in input resistance, we additionally normalized the sag amplitude to the initial peak deflection. Sag is therefore expressed as a percentage of the peak response. This normalization reduces the influence of variability in membrane resistance and allows for a more robust comparison between cell populations. Importantly, the difference in sag between the two populations remained significant when using this normalized metric.

3. Our observations indicate that repetitive prolonged 250 ms light stimulation significantly alters the release probability of ChrimsonR-expressing axons. Was there any evidence that the amplitude of the 10 ms red light responses changed across these repetitive trials? If so, what measures were taken to control this?

We used a minimum inter-sweep interval of 10 seconds to prevent cumulative changes in excitability or synaptic efficacy. We have now quantified the stability of ChrimsonR-evoked responses to 10 ms red light pulses across trials. For each cell, we performed a linear fit of EPSC amplitude across repeated 10 ms trials and found the average slope to be -0.0065, which was not significantly different from zero ($p = 0.7686$). This analysis confirms that the amplitude of 10 ms responses remained stable across the trial block, suggesting that release probability did not systematically change over the course of recording.

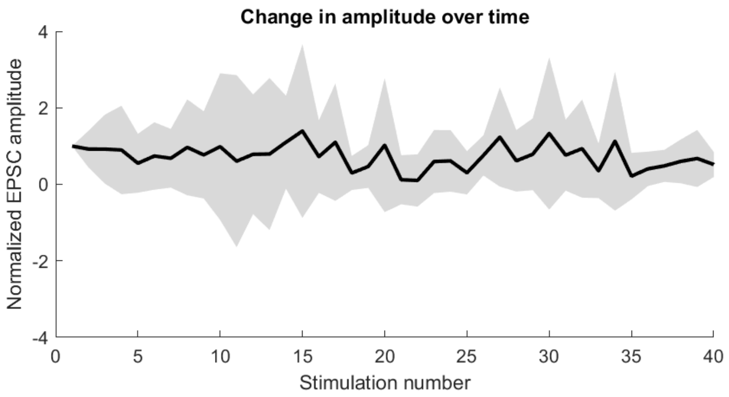

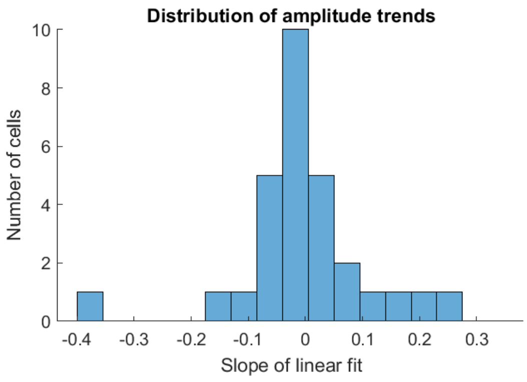

4. The description of how short-term dynamics were measured is lacking details. In the methods section, the authors state they measured the ratio by dividing the amplitude of each pulse by the amplitude of the first light pulse in the train. However, many LH cells exhibit significant summation of optically evoked EPSPs, while others do not (see Figure 2, 5 and S2). How did the authors account for this summation in their measurements? Providing clarity on this is important for understanding their findings. Relating the different EPSP kinetics to the sag/Ih difference highlighted in the manuscript (Related to point 2 above) could also provide an interesting discussion point about how LH neurons integrate cortical inputs.

We have now clarified these details in the method section. To isolate short-term synaptic dynamics from membrane summation, EPSP amplitudes were computed by subtracting the voltage baseline immediately prior to each light pulse, rather than from a fixed pre-train baseline. This approach removes the contribution of accumulated depolarization and allows for a more accurate measure of synaptic change across pulses. We have added a brief discussion of how intrinsic properties such as Ih may influence integration of temporally patterned input.

5. The introduction is notably short, with almost half of it focused on presenting the results. To elevate the manuscript's significance and broaden its appeal, the authors should more effectively highlight the importance of the problem and address the critical gaps in knowledge that this research aims to fill.

We have now re-written the introduction accordingly.

Minor

6. The authors must clarify whether retrogradely labeled neurons were recorded simultaneously with non-labeled cells, or sequential. If sequential, were positive and negative cells adjacent? If positive and negative cells were not recorded simultaneously or sequentially, it is crucial to explain how differences in opsin expression were controlled for across cells, slices, and preparations. Clarification of this information is essential for understanding the validity of the results.

We have now clarified this in the Methods. Recordings comprised both simultaneously and sequentially recorded CTb^{+} and CTb^{-} neurons, with a preference for simultaneous paired or clustered recordings whenever possible. Across 43 recorded cells (27 from mPFC- and 16 from IC-injected animals), 28 were from slices containing both CTb+ and CTb− cells (16 recorded simultaneously, 12 sequentially), 14 were from other slices of the same animals, and one was a CTb+ cell from a single mPFC-injected animal.

The average distance between adjacent CTb+ and CTb- cells was 232 ± 139 µm (mean ± SD). Overall, CTb+ and CTb- neurons were recorded from similar locations on slices (ML location, p= 0.3711 and DV location, p = 0.8115). We accounted for variability across cells, slices, and animals using a generalized linear mixed model with group as a fixed

effect and recording and slice (nested within animal/date) as random effects. This approach controls for differences in opsin expression, slice quality, and recording conditions, strengthening the validity of our conclusions.

7. The authors used AAV1 in their experiments, yet it is well-established that AAV1 possesses significant anterograde transsynaptic spread capabilities. Did the authors detect any evidence of anterograde expression of opsins in the recorded cells, even if it was only subtle? Commenting on this issue in the manuscript is important.
AAV1 spreads anterogradely at high viral titers (~10^{13} vg/ml; Zingg et al 2017). We used a diluted viral concentration (~10^{12} vg/ml), reported to eliminate anterograde spread (Zingg et al 2017). Consistent with this, we did not observe labeled cell bodies outside the injection site in our histology datasets (Fig 5). We have added this information in the Methods.

8. The LH comprises several neuronal subtypes, including excitatory and inhibitory neurons, and yet this is not mentioned in the manuscript. A better discussion of these various cell types and their potential relationships to the observed results would greatly improve the manuscript.
We have now added discussion of LH cell types in the Introduction and Discussion.

9. For the dual-opsin experiments, the methods state that amplitude comparisons were done by comparing the 10 ms light pulse responses, which is the correct approach for analyzing these data. However, in Figures 4B-D, it appears that a comparison is being made between the 590 nm, 250 ms light pulse and the 470 nm, 10 ms light pulse. This raises a critical question: Were these data analyzed differently from those presented in Figure 3? If so, such comparisons could undermine the results. If not, it is critical to revise the presentation of these data to prevent confusion. Clearer communication and presentation are important.
We have now clarified this presentation - thank you for raising this. In the original analysis (orginally Fig. 4B-D, now Fig. 8C-E), amplitude comparisons were based on 10 ms light pulses, apart from a subset of neurons (n = 8), where the response to 10 ms red light stimulation was not reliably detected, while a small but consistent response was observed during the 250 ms red light pulse. These neurons were distributed across both the mPFC-preferring and IC-preferring categories. We recognize that this was not clearly explained. To standardise this analysis, we now only use the ChrimsonR responses evoked by the 250 ms red pulse for Fig. 8 cell classification, yielding the same categorization of neurons and the same overall conclusions as the original analysis. For full transparency, we report the comparisons of 10 ms and 250 ms responses below (Wilcoxon signed rank test, n=31):

Amplitude -32.72 ± 53.66 pA vs. -41.92 ± 70.67 pA, p=0.1183.
Latency 4.03 ± 1.56 ms vs. 3.44 ± 1.99, p=0.1710.
Rise time 2.54 ± 1.39 ms vs. 1.94 ± 1.28, p=0.1047.
Decay time 9.70 ± 8.18 vs. 6.85 ± 4.28, p=0.3794.

10. In multiple brain regions, it has been established that excitatory cells in layers 5 (L5) and 6 (L6) display distinct synaptic properties. Could the authors provide insights on whether the short-term dynamics observed are influenced by the ratio of L5 to L6 cells labeled in these specific brain regions? Adding a discussion point on this topic would also be helpful.
We have now added a discussion point on a possibility of layer differences. However, we are unaware of papers that have looked at dynamics of insula L5 v L6 output synapses. We are aware of PFC work from Adam Carter and colleagues, Solange Brown's work on

L6 sublaminar differences in S1, Matthew Larkum's results on L5 v L6b in S1, and the Allen Institute data in V1, which however did not show robust or consistent L5 v L6 differences.

11. Figure 2 emphasizes the modest short-term depression of mPFC inputs to LH neurons (Figure 2 G), and yet the AP probability increases with spike number. These two pieces of data don't necessarily match. Can the authors elaborate on these data? Is there enough evidence to suggest there are two types of mPFC synaptic responses (facilitating and depressing)? For example, the progressive pulse ratio plots in Figure S2 seem to highlight two types of responses for mPFC and perhaps others. This could relate to the fact the authors observe both L5 and L6 labeled (related to point 10 above).
There is indeed a minority of mPFC EPSPs showing facilitation. We made this more explicit in the revised manuscript by using the terms 'mostly non-facilitating' rather than 'depressing'. To test if progressive pulse ratio correlated with AP probability, we added Fig. 3H where no effect was observed.

12. The horizontal lines above Figure 4 F and J seem unnecessary
Removed.

13. The titers of the viruses used is not reported; despite saying it is in Table 1
Titers of viruses are now included in Table 1.

14. What was the N for the mPFC data in Figures S4.
Sample size information has been added in the revised text for this new Fig. 5. For this anterograde tracing experiment the sample size was 3 mice for mPFC and 3 mice for IC.

15. Figure 5G says 11 of 11 mPFC cells, but the text in the results says 10/10 cells.
Apologies for this error. We have now carefully checked all proportions and fixed inconsistencies.

16. Discussion, paragraph 3: the term "powerful" is overstated given the overall weak nature of the inputs (<5 mV) and low probability of driving spiking activity in LH cells.
The text has been revised to remove the term "powerful" and describe the inputs more accurately without overstatement.

17. Figure 4 legend: The description for panel A and C refers to seeing figure 4. I think the authors mean figure 3.
Edited accordingly.

18. Scales are missing values or are not reported in the legend for several figures.
Scales are now reported in legends of all figures.

19. Testing the dual-opsin interactions over cell bodies is not the same as testing over axons/terminals and it is unclear why these experiments were done and what they add.
This adds information about the spectral separation with different irradiances at the most sensitive compartment, i.e. AP firing when activating most of the dendritic, somatic and axon-initial segment membrane. We feel this is important particularly because it shows Chronos is minimally engaged by 590 nm light (Fig. 6C,D). We have made this clearer in the text.

20. The references were not displayed throughput the manuscript, making it challenging and annoying to check references.

We sincerely apologise for this file conversion issue, and will double-check visibility in the generated pdf before submitting.

Referee #2:

In this study the authors employed anatomical tracing and optogenetic circuit mapping in mice to identify and characterise distinct excitatory cortical (including subicluar and hippocampal) inputs to the lateral hypothalamus (LH), with a particular focus on the medial prefrontal cortex (mPFC) and the insular cortex (IC). The authors show that the mPFC provides the most widespread and potent monosynaptic input, apparently followed by the IC, subiculum, and ectorhinal cortex. The study revealed that insular inputs preferentially target a highly excitable dorsolateral subpopulation of LH neurons, characterised by a pronounced h-current (Ih) and post-inhibitory rebound. In contrast, mPFC inputs extensively innervate LH neurons that project back to the mPFC, i.e reciprocally connected LH neurons. LH neurons that convergent input from mPFC and IC, simultaneous stimulation sums supralinearly, suggesting robust potential for cortical co-activation driving powerful LH output. The authors propose that these findings reveal a specialised, pathway-specific integration within the LH that supports rapid, reciprocal communication with higher-order cortical regions involved in survival behaviours.

However, while I consider this study to be very interesting, at least the writing and editing of the manuscript leaves much to be desired and is not acceptable in its present form. It is very obvious that the manuscript was initially intend for a different journal because the authors did not even make the effort to change the format to J. Physiol. style (i.e. Abstract, Introduction, Methods, Results, Discussion). Moreover, and very annoyingly so, the citation style was not altered and but not even checked because upon submission the in-text citations became incomplete so that it is difficult to refer back to the actual paper(s) cited (see my comments below). Furthermore, it is not entirely clear which findings of the study shown for the first time in this work and are not only supporting earlier studies. In particular, the retrograde tracing studies performed in this study have previously been done using other tracers and led to very similar conclusions. Here it would have been nice to seen how cholera toxin b (CTb) labels individual axons or presynaptic structures. This would have greatly enhanced the findings of the study.
We have substantially re-written the manuscript to much improved readability, clarity of added value of our study, and using J Physiol style. Our sincerest apologies for the in-line citations which seem to have been converted to white font in the submission system. We have now corrected this and formatted to J Physiol style, and will remember to double-check visibility in the generated pdf before submitting.

Specific points

In the main text body and figure legends, abbreviations are used that have not been introduced previously. Several figure legends appear incomplete or insufficiently detailed. In every legend, clarify the abbreviations, the sample sizes, and whether error bars represent SEM or SD. Provide sufficient information for readers to interpret each panel without referring back to the main text.
We have now revised the text and figure legends accordingly.

Citation style

It appears that this manuscript was previously submitted to another journal which uses a

different citation style. When converting the manuscript for submission to the Journal of Physiology, the citation style was not checked and converted, resulting in citations that are not fully legible and/or incomplete. Please convert the current numerical inline citations to a style that conforms to the conventions of this journal (e.g. 'Smith et al., 2020'). Ensure that all in-text citations correctly match the references in the reference list and that references are fully legible and consistently formatted. Furthermore, citations should use unified journal names (e.g. either Current Biology or Curr Biol but not both).
Edited accordingly.

Statistical Reporting

The rationale for using SEM instead of SD should be clarified, especially where sample sizes are small (e.g., n=4). SEM can underestimate the variability in small samples, so be sure to justify why SEM is most appropriate and how many independent animals versus slices or cells were used.
We now use SD throughout and report sample sizes as suggested.

Displaying error bars for a dataset with only two data points (as e.g. in Fig. 2) is not acceptable and any statistical comparison is unlikely to be rigorous. I suggest to simply describe the results qualitatively
For all data shown here, please discuss whether such a disparity in sample size affects the reliability of any statistical test. For example, when describing synaptic dynamics you compare in Figure 2H,I data from mPFC (e.g., 11 of 49 cells) and the ventral subiculum (3 of 4 cells), i.e. with a sample size that differs by a factor of more than 10. If you retain those analyses, justify the choice and interpret with caution.
In the revised manuscript we have left data outside of statistical comparison if the number of cells was less than 5, and only describe these qualitatively.

Results
The authors use a lot of expressions such as Latencyunconfirmed and Latencyconfirmed, Z, U, p, adj-p, χ2 (and several others) without explaining their meaning. This should be amended.
We now use descriptive statistics in the main text, and have moved the fine statistical detail into Methods, figure legends (where appropriate) and Table 3 (statistical data table).

Page 3, 5th para.
Should read either 'fibre of passage' or 'en passant fibres'.
Now corrected.

Page 4, 5th para. and Figure 2G-I
The authors state that 'Responses to these inputs displayed distinct synaptic dynamics (Fig. 2G). The paired-pulse ratio (EPSP5/EPSP1) was higher for IC and aIC inputs compared to mPFC inputs, indicating facilitation of responses to insular inputs'. However, when comparing the EPSP2/EPSP1 paired pulse ratio (PPR) it appears that the ventral subiculum response has a stronger facilitation than the aIC; in addition the pIC has a substantially lower PPR than vSub. Please clarify.
To clarify this observation, we performed additional statistical analyses on the EPSP2/EPSP1 paired-pulse ratio across the different input regions. When including all datasets, a Kruskal–Wallis test did not reveal a statistically significant difference between groups ($p = 0.054$). Although post hoc pairwise comparisons are generally not interpreted when the omnibus test is not significant, we nonetheless examined them for completeness. These comparisons showed no significant differences after correction for

multiple comparisons (vSub vs aIC: p = 0.188 uncorrected, p = 0.255 corrected; vSub vs pIC: p = 0.067 uncorrected, p = 0.301 corrected). Taken together, these analyses indicate that while the vSub responses visually appear more facilitating in the EPSP2/EPSP1 ratio, these differences are not statistically significant. We haven't changed text based on this at this point as we feel this discussion may be distracting if added to the manuscript, especially as we have now excluded datasets with <5 samples (ectorhinal cortex and dorsal/ventral subiculum) from statistical comparisons in the revised manuscript.

In this context, please describe the rationale for using the EPSP5/EPSP1 rather than the EPSP2/EPSP1 ratio.
We analysed EPSP5/EPSP1 ratio to examine how a burst of presynaptic spikes would be integrated postsynaptically, and to align with other reports on PFC long-range circuitry (e.g., Collins, Anastasiades et al 2018).

As shown in Fig. 2H,I, the sample size for the pIC, the vSub and in particular the ECT and dSub is rather low and is likely to result in an over-interpretation of data. Please consider excluding or at least separating such minimal data.
In the revised manuscript we leave these data outside of statistical comparison.
Please clarify the meaning of '100% facilitation' for the y-axis label of Figure 2I. I assume this is supposed to mean 100% of all responses show facilitation but this axis label is misleading. Please rephrase.
Thank you for pointing out this error. We have now clarified that this means the % of cells with facilitating EPSPs.

Page 4, 5th para. and Figure 2K,L: Action Potential (AP) Probability
The authors state that 'Action potential (AP) firing was only observed in response to mPFC and IC stimulation (Fig. 2J-L)'. However, no AP firing was observed for the aIC and the -IC- and pIC showed a response in one case each (panel L). For all other input regions (ECT, dSub and vSub) the sample size is limited. Please provide a statistical comparison for the data to make firm conclusions.
We have now added a note that AP firing was rare overall, and have added the statistical comparison (restricted to mPFC, -IC-, aIC and pIC), which found no significant differences in AP probability (probability of at least one AP; p = 0.251, Kruskal-Wallis test).

Figure 3B Legend
Update this legend to include a complete description of panels, labelling, sample sizes, and any color coding or arrows that appear in the figure. Indicate precisely which cortical injection sites or transgenes each panel corresponds to.
Now corrected, Fig. 7 in the revised manuscript.

Page 7, paragraphs 1-3
To these for preferential targeting of identified reciprocally connected LH neurons by mPFC and IC afferents, the authors used co-injections of Ctb and channelrhodopsin 2 (ChR2). For this, two mice for each neocortical region were used. This is quite low in particular in the light of the statement that the data suggest that 'mPFC inputs are specialised to excite reciprocally connected LH neurons'. Please indicate also the fraction of reciprocally connected LH neurons in mPFC and IC.
We should clarify first that the number of mice used for histology was two for each neocortical region while for electrophysiological experiments three (IC) and six (mPFC) further mice were used. Therefore, we feel the conclusion about mPFC is justified. However, we have removed statistical testing on the IC connection amplitudes due to the small sample and corrected the statements about IC to note these results are preliminary

due to the low sample size. The fraction of reciprocally connected neurons in each case are noted in the manuscript (11/11 for mPFC, and 2/6 for IC).

Figure 5
In panel C, the x-axis label (AP position) should be clarified. In addition, the y-axis label 'CTb counts' is somewhat misleading and probably means no. of CTb-positive neurons. Please change.
In panel E, please clarify what the example traces show.
Now corrected, Fig. 9 in the revised manuscript.

Discussion

Labelling Data

While the retrograde tracing results are consistent with prior studies (e.g., Hosoya & Matsushita, 1980; Kita & Oomura, 1982), the manuscript should more explicitly highlight what is novel in these new experiments. Emphasise how your findings on subregional or cell-type-specific targeting go beyond older data. Otherwise, the impression is that the labelling results replicate mostly 'known' pathways.
We now note that most of the prior studies were in a different species (rat), and emphasise our finding of the layer specificity and continuity of the labelled populations.

Description of Supralinearity

It would be helpful to explain whether the observed supralinear integration depends on particular stimulation intensities or frequencies. Are there parameters that prevent a supralinear summation when mPFC and IC inputs are co-activated? If the data suggest that even modest co-activation triggers a supralinear response, clarify this explicitly.
We did not systematically vary intensity or frequency within the dual-opsin experiments, so conditions under which supralinearity might fail remain unknown. However, testing frequencies would be limited because the 250 ms pulses required to isolate the Chronos response limits the use of higher frequencies. Supralinear integration was consistently observed under our stimulation parameters, using modest 10 ms pulses with a 10 s inter-sweep interval. Saturated intensities for ChrimsonR and Chronos were used to account for their partial spectral overlap; without saturation, dual-light stimulation could simply increase the ChrimsonR response rather than reveal supralinear summation. Control experiments with a ChrimsonR-only dataset confirm that this approach effectively isolates each input.

Provide a more detailed explanation of how the 'sum versus dual stimulation' ratio was computed, and note any conditions under which that ratio might not exceed 1.0.
We have now clarified in the Methods that this metric was calculated by dividing the response amplitude to simultaneous stimulation by the sum of the response amplitudes evoked by stimulation of each input individually. Ratios greater than 1 indicate supralinear integration, whereas ratios equal to or below 1 reflect linear or sublinear summation. In a subset of neurons that responded only to ChrimsonR stimulation, the ratio did not exceed 1 (new Fig. 7M-O).

In the discussion, it would be helpful if you could suggest some potential mechanisms for the supralinear summation of mPFC and IC inputs and describe them in sufficient detail as this is an important and novel finding.

Edited accordingly.

Page 8, 4th para.
You mention specifically that '... post-inhibitory rebound makes these neurons particularly responsive after subcortical inhibition, possibly from topographically colocalized inputs from the central amygdala, which could modulate their activity depending on timing ...'. This is an interesting point. However, I suggest to briefly explain the mPFC-LH-CA neuronal circuitry and the evidence is for macro- and/or microscopic colocalisation of these inputs.
Edited accordingly.

Should either read Ih of h current or HCN channel current.
Changed to h-current.

Page 8, 5th para.

What is meant by 'canonical' LH neuron subtypes?
We have now removed this colloquialism.

Abbreviations such as MCH and Orx neurons for Melanin-concentrating hormone and Orexin neurons, respectively should be introduced or not used at all if they appear only once.
Edited accordingly.

Dear Dr Karnani,

Re: JP-RP-2026-288596R1 "**Integration of cortical inputs in the lateral hypothalamus is dominated by the medial prefrontal cortex**" by Lotte Razenberg, Pieter Nicolaas de Greef, Huib Mansvelder, and Mahesh Miikael Karnani

Thank you for submitting your manuscript to The Journal of Physiology. It has been assessed by a Reviewing Editor and by 3 expert referees and we are pleased to tell you that it is acceptable for publication following satisfactory revision.

The referee reports are copied at the end of this email.

Please address all the points raised and incorporate all requested revisions or explain in your Response to Referees why a change has not been made. We hope you will find the comments helpful and that you will be able to return your revised manuscript within 4 weeks. If you require longer than this, please contact journal staff: jp@physoc.org.

Your revised manuscript should be submitted online using the link in your Author Tasks Link Not Available. This link is accessible via your account as Corresponding Author; it is not available to your co-authors. If this presents a problem, please contact journal staff (jp@physoc.org). Image files from the previous version are retained on the system. Please ensure you replace or remove any files that are being revised.

If you do not wish to submit a revised version of your manuscript, you must inform our journal staff (jp@physoc.org) or reply to this email to request withdrawal. Please note that a manuscript must be formally withdrawn from the peer review process at one journal before it may be submitted to another journal.

TRANSPARENT PEER REVIEW POLICY: To improve the transparency of its peer review process The Journal of Physiology publishes online, as supporting information, the peer review history of all articles accepted for publication. Readers will have access to decision letters, including Editors' comments and referee reports, for each version of the manuscript, as well as any author responses to peer review comments. Referees can decide whether or not they wish to be named on the peer review history document.

ABSTRACT FIGURES: Authors are expected to use The Journal's premium BioRender account to create/redraw their Abstract Figures. Information on how to access this account is here: https://physoc.onlinelibrary.wiley.com/journal/14697793/biorender-access.

This will enable Authors to create and download high-resolution figures. If authors have used the free BioRender service, they can use the instructions provided in the link above to download a high-resolution version suitable for publication.

The link provided should only be used for the purposes of this submission. Authors will be charged for figures created on this account if they are not related to this manuscript submission.

LANGUAGE EDITING AND SUPPORT FOR PUBLICATION: If you would like help with English language editing, or other article preparation support, Wiley Editing Services offers expert help, including English Language Editing, as well as translation, manuscript formatting, and figure formatting at www.wileyauthors.com/eeo/preparation. You can also find resources for Preparing Your Article for general guidance about writing and preparing your manuscript at www.wileyauthors.com/eeo/prepresources.

RIGOUR AND REPRODUCIBILITY: We recommend authors consult The Journal's Rigour and Reproducibility requirements to ensure that their manuscript is suitable for publication: https://physoc.onlinelibrary.wiley.com/pb-assets/hub-assets/physoc/documents/TJP-Rigour-and-Reproducibility-Requirements.pdf

REVISION CHECKLIST:

Check that your Methods section conforms to journal policy: https://jp.msubmit.net/cgi-bin/main.plex?form_type=display_requirements#methods

Check that data presented conforms to the statistics policy: https://jp.msubmit.net/cgi-bin/main.plex?form_type=display_requirements#statistics

Upload a full Response to Referees file. To create your 'Response to Referees' copy all the reports, including any comments from the Senior and Reviewing Editors, into a Microsoft Word, or similar, file and respond to each point, using font or background colour to distinguish comments and responses and upload as the required file type.

Please upload two versions of your manuscript text: one with all relevant changes highlighted and one clean version with no changes tracked. The manuscript file should include all tables and figure legends, but each figure/graph should be uploaded as separate, high-resolution files. The journal is now integrated with Wiley's Image Checking service. For further details, see: https://www.wiley.com/en-us/network/publishing/research-publishing/trending-stories/upholding-image-integrity-wileys-image-screening-service

You may also upload:

- 'Potential Cover Art' for consideration as the issue's cover image
- Appropriate Supporting Information (Video, audio or data set: see https://jp.msubmit.net/cgi-bin/main.plex?form_type=display_requirements#supp).

We look forward to receiving your revised submission.

If you have any queries, please reply to this email and we will be pleased to advise.

Yours sincerely,

Nathan Schoppa
Senior Editor
The Journal of Physiology

REQUIRED ITEMS

- You must start the Methods section with a paragraph headed Ethical approval (https://jp.msubmit.net/cgi-bin/main.plex?form_type=display_requirements#methods).

Research must comply with The Journal's policies regarding animal experiments (https://physoc.onlinelibrary.wiley.com/hub/animal-experiments) and adherence to these policies must be stated in the manuscript.

Authors should confirm in their Methods section that their experiments were carried out according to the guidelines laid down by their institution's animal welfare committee, including an ethics approval reference number. The Methods section must contain a statement about access to food, water and housing, details of the anaesthetic regime: anaesthetic used, dose and route of administration, and method of killing the experimental animals.

EDITOR COMMENTS

Reviewing Editor:

Methods Details:
This has been provided by reviewer#3 already. Authors need to respond to these requests.

Ethics Concerns:
Please consult O'Halloran (2024) https://physoc.onlinelibrary.wiley.com/doi/full/10.1113/JP286666 and ensure you are compliant with reporting requirements of The Journal concerning the use of animals in research.

Comments to the Author (Required):
The authors have done a good job of responding to previous critiques. However, the additional essential details pertaining to ethics and welfare are required. Please consult O'Halloran (2024) https://physoc.onlinelibrary.wiley.com/doi/full/10.1113/JP286666 and ensure compliance with reporting requirements of The Journal concerning the use of animals in research.

Senior Editor:

C
Thank you for submitting your revised manuscript to Journal of Physiology. It has been looked at by the two reviewers of the original manuscript along with the reviewing editor (RE), who all felt the additional analyses and clarifications/changes in the text resulted in a significantly improved study. The work is also considered to be quite impactful. The manuscript is now considered acceptable for publication pending the authors addressing several concerns in the area of animal ethics and procedures that were brought up by Referee 3, who is JP's Animal Ethics Editor. The authors should address all concerns that he raised and follow all of JP's reporting requirements in the area of animal ethics and welfare.

.

REFEREE COMMENTS

Referee #1:

The authors have addressed all of my previous concerns. In addition, they have performed several additional analyses/experiments that strengthen this study. The revised manuscript is significantly better

Referee #2:

The manuscript has been thoroughly revised, significantly improving its readability. All of my concerns have been addressed in detail.

Referee #3:

Thank you for submitting your manuscript to The Journal of Physiology. Some additional essential details pertaining to ethics and welfare are required.

Please consult O'Halloran (2024) https://physoc.onlinelibrary.wiley.com/doi/full/10.1113/JP286666 and ensure you are compliant with reporting requirements of The Journal concerning the use of animals in research.

1. You must start the Methods section with the subheading "Ethical approval". Include detail of the institutional approval code/number associated with the study.

2. Include the age range of the animals used.

3. "Surgeries". Give details on how an adequate depth of anaesthesia was determined to permit surgical access. Include the duration of the surgeries and indicate whether supplemental doses were required and how this was assessed.

4. Give detail of the concentration of carprofen in the drinking water.

5. "Histology". Give detail of the terminal dose of pentobarbital. Did this cause death (or deep anaesthesia), or was death caused by transcardial perfusion (exsanguination)? You must include a statement on how an adequate depth of anaesthesia was determined, if appropriate, to permit surgical access.

6."Brain slices". Give details of the concentration of isoflurane and the carrier gas.

END OF COMMENTS

Many thanks for the comments. All requested clarifications regarding animal ethics, anaesthesia, and welfare reporting have now been incorporated into the revised Methods section in accordance with journal requirements. We've submitted a new manuscript text and tracked changed document. In addition, our responses to reviewer 3's specific points below.

EDITOR COMMENTS

Reviewing Editor:

Methods Details:
This has been provided by reviewer#3 already. Authors need to respond to these requests.

Ethics Concerns:
Please consult O'Halloran (2024) https://physoc.onlinelibrary.wiley.com/doi/full/10.1113/JP286666 and ensure you are compliant with reporting requirements of The Journal concerning the use of animals in research.

Comments to the Author (Required):
The authors have done a good job of responding to previous critiques. However, the additional essential details pertaining to ethics and welfare are required. Please consult O'Halloran (2024) https://physoc.onlinelibrary.wiley.com/doi/full/10.1113/JP286666 and ensure compliance with reporting requirements of The Journal concerning the use of animals in research.

Senior Editor:

C
Thank you for submitting your revised manuscript to Journal of Physiology. It has been looked at by the two reviewers of the original manuscript along with the reviewing editor (RE), who all felt the additional analyses and clarifications/changes in the text resulted in a significantly improved study. The work is also considered to be quite impactful. The manuscript is now considered acceptable for publication pending the authors addressing several concerns in the area of animal ethics and procedures that were brought up by Referee 3, who is JP's Animal Ethics Editor. The authors should address all concerns that he raised and follow all of JP's reporting requirements in the area of animal ethics and welfare.

REFEREE COMMENTS

Referee #1:

The authors have addressed all of my previous concerns. In addition, they have performed several additional analyses/experiments that strengthen this study. The revised manuscript is significantly better

Referee #2:

The manuscript has been thoroughly revised, significantly improving its readability. All of my concerns have been addressed in detail.

Referee #3:

Thank you for submitting your manuscript to The Journal of Physiology. Some additional essential details pertaining to ethics and welfare are required.

Please consult O'Halloran (2024) https://physoc.onlinelibrary.wiley.com/doi/full/10.1113/JP286666 and ensure you are compliant with reporting requirements of The Journal concerning the use of animals in research.

1. You must start the Methods section with the subheading "Ethical approval". Include detail of the institutional approval code/number associated with the study.
We have updated the Methods section to include the institutional approval code under the “Ethical approval” subheading.

2. Include the age range of the animals used.
We have added the age range (3-20 weeks) of the animals to the Methods section.

3. "Surgeries". Give details on how an adequate depth of anaesthesia was determined to permit surgical access. Include the duration of the surgeries and indicate whether supplemental doses were required and how this was assessed.
We have expanded the Methods section to include details on anaesthesia monitoring, surgery duration, and perioperative care.

4. Give detail of the concentration of carprofen in the drinking water.

We have added the concentration of carprofen (0.067 mg/mL) to the Methods sections.

5. "Histology". Give detail of the terminal dose of pentobarbital. Did this cause death (or deep anaesthesia), or was death caused by transcardial perfusion (exsanguination)? You must include a statement on how an adequate depth of anaesthesia was determined, if appropriate, to permit surgical access.
We have clarified the protocol, including dose of pentobarbital, assessment of anaesthetic depth, and cause of death.

6."Brain slices". Give details of the concentration of isoflurane and the carrier gas.
We have clarified the concentration and delivery method of isoflurane anaesthesia.

____

END OF COMMENTS

Dear Dr Karnani,

Re: JP-RP-2026-288596R2 "**Integration of cortical inputs in the lateral hypothalamus is dominated by the medial prefrontal cortex**" by Lotte Razenberg, Pieter Nicolaas de Greef, Huib Mansvelder, and Mahesh Miikael Karnani

We are pleased to tell you that your paper has been accepted for publication in The Journal of Physiology.
- Please include a full title page as part of your main article (Word) file, which should contain the following: title, authors, affiliations, corresponding author name and contact details, keywords, and running title.

NOTE- Please include a full title page as part of your main article (Word) file, which should contain the following: title, authors, affiliations, corresponding author name and contact details, keywords, and running title.

The last Word (or similar) version of the manuscript provided will be used by the Production Editor to prepare your proof. When this is ready you will receive an email containing a link to Wiley's Online Proofing System. The proof should be thoroughly checked and corrected as promptly as possible.

Authors should note that it is too late at this point to offer corrections prior to proofreading. Major corrections at proof stage, such as changes to figures, will be referred to the Editors for approval before they can be incorporated. Only minor changes, such as to style and consistency, should be made at proof stage. Changes that need to be made after proof stage will usually require a formal correction notice.

All queries at proof stage should be sent to: TJP@wiley.com.

Following copyediting and typesetting the accepted version will be published online.

Yours sincerely,

Nathan Schoppa
Senior Editor
The Journal of Physiology

IMPORTANT POINTS TO NOTE FOLLOWING ACCEPTANCE OF YOUR PAPER:

• IMPORTANT NOTICE ABOUT OPEN ACCESS: To assist authors whose funding agencies mandate immediate public access to published research findings, The Journal of Physiology allows authors to pay an Open Access (OA) fee to have their papers made freely available immediately on publication.

The Corresponding Author will receive an email from Wiley with details on how to register or log in to Wiley Authors where you will be able to place an order.

• You can check if your funder or institution has a Wiley Open Access Account here:
https://authors.wiley.com/author-resources/Journal-Authors/open-access/author-compliance-tool.html

• TRANSPARENT PEER REVIEW POLICY: To improve the transparency of its peer review process, The Journal of Physiology publishes online as supporting information the peer review history of all articles accepted for publication. Readers will have access to decision letters, including Editors' comments and referee reports, for each version of the manuscript as well as any author responses to peer review comments. Referees can decide whether or not they wish to be named on the peer review history document.

• You can help your research get the attention it deserves! Check out Wiley's free Promotion Guide for best-practice recommendations for promoting your work at: www.wileyauthors.com/eeo/guide. You can learn more about Wiley Editing Services which offers professional video, design, and writing services to create shareable video abstracts, infographics, conference posters, lay summaries, and research news stories for your research at: www.wileyauthors.com/eeo/promotion.

• If you would like to receive our 'Research Roundup', a monthly newsletter highlighting the cutting-edge research published in The Physiological Society's family of journals (The Journal of Physiology, Experimental Physiology, Physiological Reports, The Journal of Nutritional Physiology and The Journal of Precision Medicine: Health and Disease), please click this link, fill in your name and email address and select 'Research Roundup':
https://www.physoc.org/journals-and-media/membernews

EDITOR COMMENTS

Reviewing Editor:

The authors have done an excellent job of responding to the previous critiques

Senior Editor:

Congratulations! You have addressed all of the remaining concerns around animal ethics and useage, and your manuscript is now acceptable for publication.
